# Model for successful development and implementation of Cyber Security Operations Centre (SOC)

**DOI:** 10.1371/journal.pone.0260157

**Published:** 2021-11-19

**Authors:** Maziana Abd Majid, Khairul Akram Zainol Ariffin

**Affiliations:** 1 Malaysian Administrative Modernisation and Management Planning Unit, Federal Government Administrative Centre, Putrajaya, Malaysia; 2 Universiti Kebangsaan Malaysia, Bangi, Selangor, Malaysia; Universiti Pertahanan Nasional Malaysia, MALAYSIA

## Abstract

Cyberattacks have changed dramatically and have become highly advanced. This latest phenomenon has a massive negative impact on organizations, such as financial losses and shutting-down of operations. Therefore, developing and implementing the Cyber Security Operations Centre (SOC) is imperative and timely. Based on previous research, there are no international guidelines and standards used by organizations that can contribute to the successful implementation and development of SOC. In this regard, this study focuses on highlighting the significant factors that will impact and contribute to the success of SOC. Simultaneously, it will further design a model for the successful development and implementation of SOC for the organization. The study was conducted quantitatively and involved 63 respondents from 25 ministries and agencies in Malaysia. The results of this study will enable the retrieval of ten success factors for SOC, and it specifically focuses on humans, processes, and technology. The descriptive analysis shows that the top management support factor is the most influential factor in the success of the development and implementation of SOC. The study also contributes to the empirical finding that technology and process factors are more significant in the success of SOCs. Based on the regression test, the technology factor has major impact on determining the success of SOC, followed by the process and human factors. Relevant organizations or agencies can use the proposed model to develop and implement SOCs, formulate policies and guidelines, strengthen human models, and enhance cyber security.

## Introduction

The evolution of a borderless world through cyberspace has changed the social and technological perspectives of the world. The advancement of cyberspace through the Internet has brought economic growth without barriers to entrepreneurship and enabled users to communicate and collaborate [1, 2]. This statement is also supported in [3] which stated that cyberspace has penetrated public sector organizations, private sector organizations, industries, geographical locations, and international borders. Based on the definitions stated in [4], it can be concluded that cyberspace has connected people of different worlds regardless of individuals or organizations to communicate casually or in business. Indirectly, cyberspace has eradicated boundaries between humans from various geographical locations. Public sector organizations, in particular, have been given the mandate to create a range of services online to transform the delivery of government services, boost the national economy, and enhance the well-being of the people. Data and information are of utmost importance in line with the current digital age because they enable stakeholders to make data-driven decisions, revolutionize employment patterns, and grow their businesses.

According to statistics released by Internet World Stats, Internet users as of March 2021 have reached 5,168,780,607, representing 65.6% of the world’s population. This percentage has shown that more than half of the world’s population uses cyberspace for various purposes. Malaysia registered a total of 27.43 million users, representing 84.2% of the Malaysian population. It shows a significant and sharp increase in Internet usage for Malaysia compared to the year Internet was introduced in Malaysia [5]. This growth is correlated with trends in technology development, such as open data, public data, various online transactions, social networks, and ICT systems. According to [6], a world driven by the latest technologies has put information security and user data privacy at risk. The global population’s reliance on cyberspace has exposed its users to various risks and threats of cyberattacks by irresponsible parties.

The study by [7] states that cyber threats and attacks began as early as the 1960s. In the early days of cyberattacks, it was only intended to test hackers’ self-esteem and gain recognition. However, it has been proven that threats and cyberattacks can impact victims in various ways, such as financial loss, impaired image, denial of service, and more. The techniques and procedures used in cyberattacks are increasingly challenging to detect and eliminate [8]. Many real cases of cyberattacks have resulted in millions of losses, with some reaching USD billion [9]. These facts illustrate how committed hackers are in launching cyberattacks. There are various ways to overcome these challenges, and one of the most popular solutions is the cyber security operations centre (SOC) [10]. The SOC represents a central protection group that concentrates on managing cyber security incidents through monitoring, detecting, investigating, analyzing, and preventing malicious activities. It also comprises human, process, and technology to help the organization strengthen security awareness, address compliance issues, and manage threats [11, 12].

Based on previous research, SOC has been developed and implemented by organizations without adhering to specific guidelines or international standards. Besides, past research has also highlighted that no international guidelines and standards have been used by organizations to develop and implement the SOC [13]. This is reflected in the inequality and diversity of SOC infrastructure and its implementation [14]. Given this scenario, it is not easy to measure the success of the SOC because no benchmark model can be applied. In this regard, a cross-examination of current SOC development and implementation needs to be performed to determine the factors that contribute to the success of SOC implementation.

At the same time, there is no model that outlined the basic needs of the identified success factors and incorporated them to ensure the successful development and implementation of the SOC. Thus, this study aims to identify the significant factors that contribute to the success of the development and implementation of SOC. Then, based on these factors, a proposed model is introduced to represent the essential requirement of SOC.

The main contributions of this paper are summarized as follows:

1. A conceptual model for the SOC is proposed. This conceptual model is based on previous studies that have focused on the success factor of SOC. Thus, highly significant factors were identified and included in the conceptual model from the comparison study.
2. The significance of the critical factors for the success of SOC was evaluated through a questionnaire. The questionnaires were divided into four components to obtain the requirements for developing the model for the development and implementation of SOC. The respondents for this questionnaire were cyber security practitioners from 25 ministries and agencies in Malaysia. In the evaluation, the findings from the questionnaire were analyzed using descriptive, correlation, and regression tests.
3. A model for the successful development and implementation of the SOC is proposed. This model encompasses the human, process, and technology as critical factors, with top management support, financial, and continuous improvement as secondary factors. Relevant organizations or agencies can use the model to develop and implement SOCs, formulate policies and guidelines, strengthen human models, and enhance cyber security.

The rest of the paper is organized as follows: Section 2 presents the evolution of cyberattacks throughout the year. The discussion of the success factors in implementing the SOC from previous studies is described in detail in Section 3, and Section 4 outlines the conceptual model of the SOC. Section 5 explains the instruments used in the questionnaire and the pilot test for the survey. The descriptive analysis is highlighted in Section 6, and the correlation and regression analysis are discussed in Section 7. The proposed model for the success of the development and implementation of the SOC is illustrated in Section 8. Finally, conclusions and future works are presented.

## Evolution of cyberattacks and organization preparation

Cyberattack is not something new, but it started as early as the 1960s with hacking activities on the frequency of telephone systems [7]. Since then, the concept of cyberattacks has become increasingly popular and has evolved as technology advances. The 1970s until the 1990s highlighted that cyberattacks were aimed at honing technical skills, seeking recognition, showing courage and skill, and hobby activities. During that time, the attackers were known as “Script Kiddies” or unprofessional cyber attackers. As such, it began with the creation of malware for various purposes, such as interfering with computer operations, collecting sensitive and classified information, or obtaining unauthorized access to computer systems. Malware is a collective term for many types of malicious software, such as computer viruses, ransomware, worms, and spyware. According to a study conducted by [15], in 1986, the malware was loaded into a floppy disk that would infect any computer. At this time, the malware was not intended to destroy or damage the victim’s property, but rather to prove the skills possessed by hackers and gaining recognition.

By the 2000s, the emergence of various electronic trading and shopping activities had changed the perspective of hackers to target large-scale financial gains by implementing more organized cybercrime [16]. [17] stated that exploring cyberattacks and malicious activities are becoming more sophisticated and targeted to a specific organization or system. Previous studies have also noted that the most critical cyber threat to businesses, governments, and individuals is the existence and distribution of malware. In 2000, the spread of malware, such as the LoveLetter worm known as ILOVEYOU, exploited the vulnerabilities inherent in the Windows operating system. It spreads through email using the LoveLetter subject and has resulted in a loss of USD 9 billion over a month worldwide. Later, in 2003, a malware called Slammer actively attacked the computer system [18]. Unlike LoveLetter, Slammer spreads through memory processes and infects computers connected to the network. The malware acts to cope with network traffic, which eventually causes many network packets to be lost, thus disabling the computer system. The estimated loss of the Slammer virus attack is estimated to reach USD 1 billion within five days.

The escalation of cyberattacks is a global issue facing by the world’s population. To date, the purpose and motives of cyberattacks have evolved based on various issues, such as political, revenge, monetary, and destruction aimed at specific targets [19, 20]. [3, 13] further outlines that cyberattacks compromise the confidentiality, integrity, and availability of the victim’s information. At the same time, victims suffer damages for possible loss or damage to data, disruption to business or service, loss of revenue, and even damage to ICT hardware. Additionally, this statement is supported by [21], who reported that almost 50% of cyberattacks are intended to damage the organization or individual.

The current evolution of cyberattacks applies advanced techniques and malware to launch attacks and protect themselves from being detected. It has become alarming for organizations today, as their system vulnerabilities are being exploited by these cyberattacks, especially in terms of humans, processes, and technology [22–24]. Therefore, every organization needs to increase its readiness to face the growing threat of cyberattacks. Organizations need to ensure that existing cybersecurity protections can detect, alert, protect, and prevent cyberattacks.

At the same time, cyberattacks are rampant in Malaysia. An increasing number of cyberattacks in Malaysia were also acknowledged by the National Cyber Security Agency (NACSA), as it reported that there were significant increases in cyberattacks targeting various organizations in Malaysia. Among the cyberattacks that occurred, the most dominant attacks were obstruction of service delivery, phishing, and malware. These cyberattacks resulted in loss of information, disruption of service, and compromised information integrity. Over the last ten years, the increase in incidents has been in line with the increasing number of Internet users in Malaysia [25].

Cyber security responses are divided into two mechanisms; through internal and external approaches [6]. The external method refers to the mechanism that is taken at the national and global levels. At a national level, responsibility is addressing the issue of cyberattacks. Therefore, legislation and regulation of cybercriminals’ offenses are one of the ways to manage and prevent widespread cyberattacks [26].

As for the organizational level, the work by [27] has outlined the mechanisms to protect the information security infrastructure through a study of ten organizations that have adopted cyber security strategies. The work suggests that the organization can prevent cyberattacks through technological means (e.g., access control, software control) and non-technological approaches, such as applying policy and agreement. Additionally, it also supports the cyber security detection mechanism as an effective operational strategy for identifying cyberattacks. Once a cyberattack has been identified, the organization can implement appropriate responses and corrective actions.

According to [28, 29], organizations need to strengthen their security infrastructure to ensure that they are robust and resilient to counter cyberattacks. The reciprocal activities that an organization can perform are to conduct a continuous risk assessment, implement preventive maintenance regularly, and establish policies and procedures to protect information security. In addition, it highlights the importance of implementing three security measures, namely, prevention, detection, and corrective controls, to enhance the cyber security level of the organization. The organization can achieve these three security measures by establishing an SOC [30].

These findings and past studies on the evolution of cyberattacks are essential as the basis for establishing SOCs to monitor and control cyber incidents. SOC is generally an operation centre for continuously identifying, managing, and monitoring cyberattacks or threats to an organisation’s infrastructure and applications. It is intended to safeguard the confidentiality, integrity, and availability of information. It is built up from the availability of skilled people, the right technology, and the right processes. With advancements in technology, cyberattacks cannot be prevented entirely, but organizations can take appropriate steps to respond appropriately in the event of an attack [31].

## Success factors for the implementation of SOC

Although organizations are free to implement SOCs in their way, they can refer to past research on the success factors of an SOC. These studies can guide organizations to ensure that SOC implementation is in a proper context and sufficient. The SOC success factors based on previous studies are presented in Table 1.

**Table 1:** Success factors for SOC.

Study	Top Management	Financial	Strategy	Human	Process	Technology	Environment	Analysis & Report	Physical Space	Continuous Improvement
IBM [31]				1	1	1				
Ernst & Young [30]	1	1	1	1	1	1	1	1	1	1
Crowley et al. [32]	1	1	1	1	1	1	1	1	1	1
Torres [33]				1	1	1				
Schinagl et al. [14]	1	1		1	1	1				
Onwubiko [1]			1	1	1	1				1
Mansfield-Devine [34]		1		1	1	1		1		
McAfee & Intel Security [35]			1	1	1	1				1
Sundaramurthy et al. [36]				1	1	1				1
Georgiadou et al. [37]		1	1	1	1	1	1			1
Lubis et al. [38]	1	1	1	1	1	1	1	1	1	1

From Table 1, three factors have been agreed upon and selected by all prior studies as the essential factors for the success of SOC, namely human, process, and technologies. There was an evolution of the success factor from three factors in 2013 to ten factors in 2014 [30], again emphasizing the importance of these factors in 2020 [38]. While there have been changes to success factors, humans, processes, and technologies are still the key elements of successful SOC implementation. Based on previous studies, it was found that these three factors are related and complement each other to enable SOC to function effectively and productively. Coordination between humans, processes, and technologies is essential for establishing harmony between skills, systematic processes, and the technologies that is used to create strong cyber defenses to protect organizational assets. This is the fundamental reason for SOC success factors which this study is based upon. At the same time, seven out of 11 studies further agree that continuous improvement is a crucial factor influencing the success of an SOC. This factor implies that the SOC needs to continually change in line with technology advances and evolution of cyberattacks.

The importance of the human factor is also evidenced by previous research that emphasizes this element as one of the cores for SOC to function efficiently. The study by [1] defined humans as the most critical element in implementing SOC. Thus, without the human factor, the SOC cannot function fully, even with advanced technology. This statement is also supported by [11, 37], which states that it requires a group of skilled employees for the SOC to work effectively. SOC personnel must outsmart malicious attackers, determine suspicious activities, and solve cyber incidents when they occur. In addition, [10] highlights the importance of SOC in acquiring experienced and highly qualified employees in cyber security, especially in log analysis. At the same time, [30] emphasized the talented workforce and profound technical knowledge. The review by [13] has also listed nine skillsets and knowledge that are required by the employees to ensure the implementation of the SOC is well function and operated: (1) security monitoring, (2) incident handling, (3) forensics, (4) threat intelligence, (5) coding and development, (6) risk management, (7) malware analysis, (8) knowledge and skills in cyber security: penetration test and vulnerability management, and (9) network communication.

Besides technical knowledge and skills, soft skills are also crucial for SOC [32]. Communication is one of the soft skills required for SOC. It is vital that employees in the SOC need teamwork and free flow of information about operational programs. The study by [10] outlines the need to have a positive attitude and natural curiosity among the SOC’s employees to keep them informed about the new cyberattacks trend as it continues to evolve. It is also supported by [36], which states four factors that influence one’s competence: skills, empowerment, creativity, and growth.

The next element that is crucial for SOC is the process. According to [36], a process is defined as a step or procedure to achieve the desired goal. The study by [14] highlights the importance of establishing fully defined processes between the components in the SOC to ensure consistent and continuous operations. Further, fully defined processes are also necessary to determine the actions and responsibilities of the members in the SOC [33, 38]. Thus, these processes and procedures must be properly documented to ensure effective communication and facilitate changes in management. [35] recommends using a uniform template for the systematic documentation of the processes and procedures to maintain consistency. In addition, to safeguard the efficiency of the SOC, repetitive tasks should be performed automatically in SOC. However, it is essential to note that process factors always depend on the functions, services, and technologies used to establish the SOC.

Technology is also recognized as a fundamental factor in the establishment of SOC. The study by [30] encourages the organization to be equipped with the latest and appropriate technology to protect security postures. Thus, with proper tools and technologies, skillful employees can effectively understand the organization’s technical environment and solve incidents. The introduction of technology in SOC allows the process to be supported by automation. This is supported by [36] which stated that the automation process or help from the technologies will improve the operation efficiency. Such software or technology can be as sophisticated as security information and event management (SIEM) or simple scripts from a programming language [39]. The technologies ease and allow employees to engage in incident management with an in-depth investigation and provide automation for repetitive tasks.

The study by [40] distinguishes the technologies into two perspectives. The first perspective focuses on monitoring, identification, and evidence collection (e.g., through logs). Hence, it relates to the incident phase of cyber security. The pre- and post-incident phases are handled from the second perspective. It involves vulnerability scanning, penetration testing, and malware analysis. Therefore, the second perspective refers to an additional function that supports the main aspects of the SOC. A recent study by [13] summarized the requirements for technology in SOC into six groups: (1) monitoring and log collection, (2) analysis, (3) incident management and response, (4) forensic, (5) cyber threat intelligence, and (6) fundamental cyber security operational management.

Although previous studies highlight the necessity of technology for SOC, they do not individually suggest tools other than SIEM. In addition, [30] encourages the organization to consider a cost-saving approach at the beginning of its establishment. Additional tools and technologies can be added later, once the organization has stabilized. Hence, by referring to Table 1, financial is also considered one of the elements for establishing SOC.

## Conceptual model for SOC

**Fig 1:**
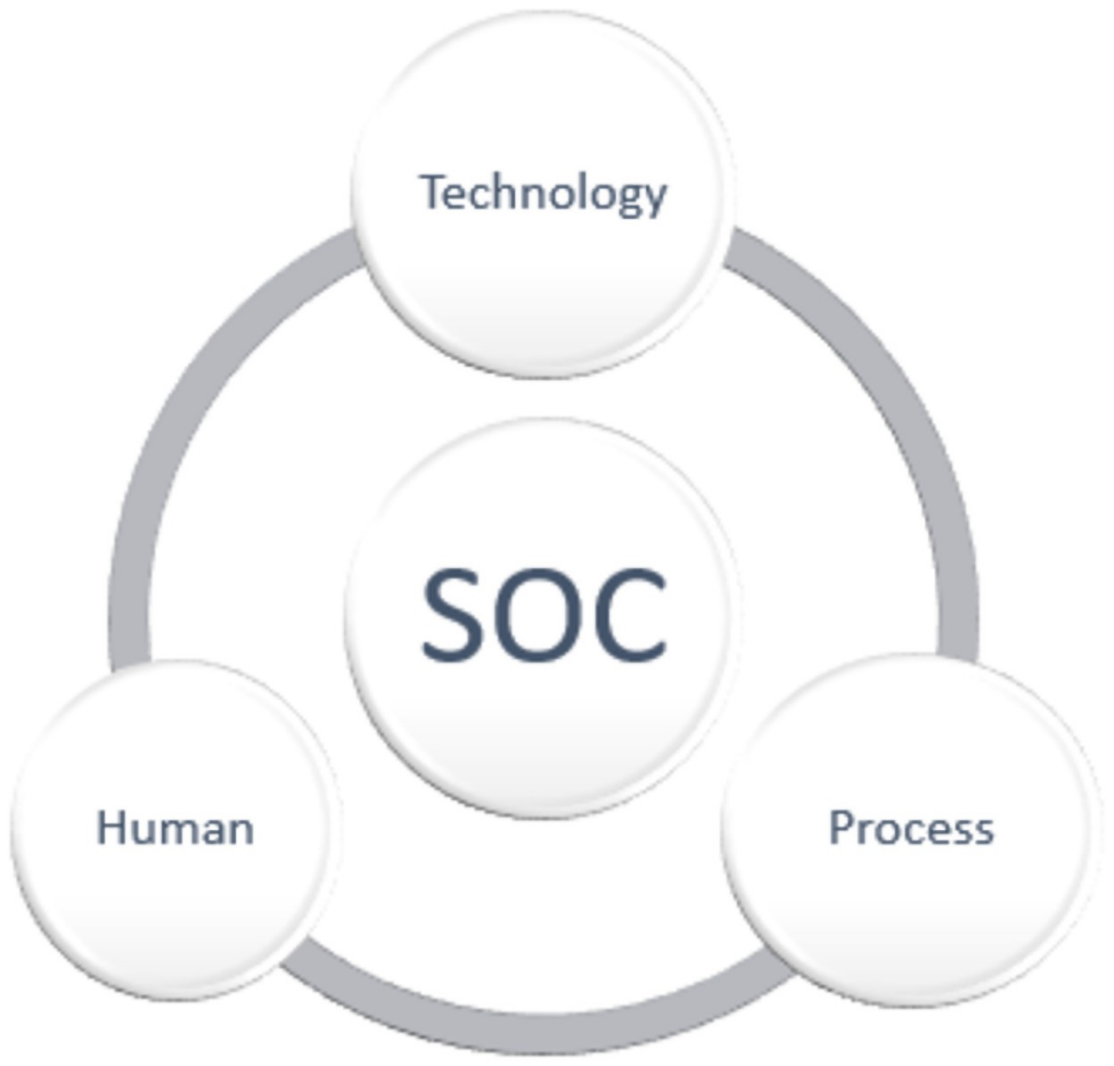
Conceptual model for the success of SOC establishment.

SOC can be considered as one of the solutions to protect the organization from cyberattacks. [14] stated that the framework of the SOC is dependent on the direction set by the organization. It can be implemented by the missions, objectives, financial, and other factors that influence the organization’s operation. Although the SOC can be developed based on organizational needs, its basic requirements and scope must be identified. These requirements cover three critical areas in cyber security: monitoring, analysis, and response. As highlighted in the previous section, human, process, and technology are crucial elements for the success of SOC establishment. The conceptual model for the successful implementation of the SOC is illustrated in Fig 1.

Fig 1 summarizes the relationship and importance of human, processes, and technology in implementing the SOC. Based on evidence from previous studies, these factors depend on the role and functionality of the SOC. With the technology application and proper established process, the employee’s motivation in SOC can be enhanced as it permits the automation of repetitive procedures. Consequently, employees have more time to explore the knowledge that requires human touch, such as threat intelligence. Thus, it can allow employees to be trained leading to advanced skilled workforce in cyber security.

Additionally, the conceptual model is supported by continuous improvement to ensure that these three elements are always relevant and up-to-date. Further, it also includes monetary aspects, as it plays a critical role in improving the organization. It is a mutual understanding that the implementation of technology requires high financial allocation. If the organization is supported with sophisticated technology but with an unskilled employee, it will leave an inefficient SOC to operate. Similarly, if the applied technology does not leverage the knowledge and skills available to employees, it will also jeopardize the implementation of SOC. Further, without a well-established process in SOC, the relationship between human and technology factors will be ineffective. Hence, selecting appropriate technology, knowledgeable, and skillful employees, and an adequately established process are critical for implementing an effective and efficient SOC. Therefore, these factors are applied as indicators in survey instruments for the establishment of SOC, especially in the Malaysian environment.

## Methodology

### Instrument for questionnaire on development and implementation of SOC

This study used questionnaire forms as instruments. The contents of the questionnaire were based on the conceptual model described in the previous section. Its objective is to develop a model for the successful development and implementation of SOC. It covers three elements: human, process, and technology.

The questionnaire forms were divided into two categories. The first method applied the structured (closed) and guided questionnaire, where it accepts the right or almost right answer to the statements given. The selection of this method allows analysis to be performed efficiently, accurately, and directly based on the questionnaire form. The second method uses a Likert scale to measure one (1) to five (5). A Likert scale is a common form of a questionnaire, as it can provide a reliable way of measuring opinions, perceptions, and behaviors. It also indicates the respondents’ agreement and disagreement with any statement.

Furthermore, applying the Likert scale enables more choices that will make it easy for the respondent to understand and answer the survey question [41, 42]. The scale of one (1) to five (5) is used to have a midpoint value with an odd number of options. The response scales are defined as follows: (1) strongly disagree, (2) disagree, (3) moderately agree, (4) agree, and (5) strongly agree. The survey was divided into five sections containing 81 questions, as described in Table 2.

**Table 2:** Parts in the questionnaire.

Components	Part
General information	A
Knowledge about SOC	B
Success factors of SOC	C
Involvement of human, process and technology in SOC development and implementation	D
Evaluation of organizational cyber security monitoring	E

Part A in the questionnaire is intended to obtain the general information and the organization detail of the respondent. It contains eight questions with a combination of answer choices and fills in the blanks. Part B aims to test the respondents’ knowledge of the development and implementation of SOC. It consists of 19 questions, where a nominal scale is applied as the choice of answer. The respondent selects one of the options: Yes, No or Not Sure. Part C is intended to test the respondents’ knowledge of the success factors that contribute to the development and implementation of SOC. This section consists of 17 questions. In Part D, the respondents were tested to measure their understanding of the human, process, and technology elements in the SOC. The questionnaire consists of 27 questions. Lastly, Part E is proposed to obtain the respondents’ information on cyber security strategies and approaches that have been implemented in their organization. The questionnaire consists of ten questions. The questions in parts C, D, and E were measured using a Likert scale. Table 3 summarizes the survey instruments used in the survey. The resources used in designing the question instruments in the survey were derived from previous studies, which have been discussed in previous sections.

**Table 3:** Summary of initial instruments for questionnaire.

Part	Constructive measurement
A	General information about respondent
B	General information on SOC
	SOC framework
	SOC function
	General factors for successful SOC
C	Factors for successful SOC
D	Human factor
	Process Factor
	Technology Factor
E	Cyber security monitoring

This study applies a non-probability sampling method in which samples are selected from a population simply because they are readily available to researchers and are nominated based on population characteristics and study objectives. The questionnaire forms were distributed online through the Google Survey Forms platform to Malaysian ministries and agencies. The sampling from the population for this study was based on the work of [43]. The respondents for the questionnaire represent cyber security employees from 25 ministries and agencies in Malaysia. The respondents for the questionnaire represent cyber security employees from 25 ministries and agencies in Malaysia. They are directly involved in the development and implementation of SOC in Malaysia’s public and private sectors. The overall population of this organization was 75 people. Hence, by referring to [44], 63 employees were chosen as the target population or sample size.

### Pilot study and techniques for data analysis

Once the questionnaire has been developed, it is evaluated by cyber security experts. It is intended to determine the relevance and importance of questions and to validate their content. Thus, this evaluation can produce a high-quality questionnaire by maintaining the scope, applicability, usage of appropriate language, and ease of understanding. In addition, this content validation activity by experts is in line with [45]. It stated that problems, fractions, reviews of measurement questions, and the questionnaire content need to be verified by experts in the relevant field so that improvements can be made before the actual survey is implemented. The study also suggested that the appropriate number of experts can be two, three, or up to 20. For this study, five experts were selected to evaluate the instruments: two experts from the government, two experts from the industry sector, and one from an academic background. The experts assessed two categories: the relevancy and importance of the questions. The assessment of the significance of the questions was based on the weightage provided by the experts. The definition of the weightage is divided into categories: (1) ignore, (2) low priority, (3) medium priority, and (4) high priority. The results of the evaluation by the experts are given in Table 4. After amendments, all recommendations were taken into consideration, and the number of items for the questionnaire increased to 81.

**Table 4:** Summary of expert evaluations.

Part	Evaluation
A	At least 94.3% of the experts agreed that all questions were relevant. At the same time, at least 51.4% of experts agreed that the questions in Section A were categorized as high priority.
B	At least 80% of the experts defined B1 questions as relevant and high priority. For B2, at least 53.3% agreed that the questions were highly significant, while 80% described them as relevant. For B3, at least 45% of experts thought the question was a high priority, and at least 75% of them thought it was relevant. For B4, at least 60% of experts judged the question as high priority, and at least 80% thought it was relevant.
C	At least 68% of the experts defined the questions as a high priority, and at least 98.8% of the experts agreed that they were relevant.
D	At least 72.3% of the experts defined the questions in D1 as high priority and 100% relevant. For question D2, 100% of the experts believed that the question was a high priority and relevant. For D3, at least 91.7% of the experts thought that the question was a high priority and 100% appropriate.
E	At least 94% of the questions were of high priority, and 100% of them were relevant.

A pilot test was conducted to ensure the reliability of the instrument. The pilot test was used to identify problems that arise in a questionnaire and to provide the instrument’s validity. As such, 25 respondents were chosen for the pilot test. Then, Cronbach’s alpha test was performed to measure the reliability between the items in the instrument. Cronbach’s alpha measurements use a value between 0 and 1. Hence, if the value is closer to 1, it indicates high reliability of the items. The Cronbach’s alpha test for each component of the instrument is highlighted in Table 5. The results show that all components have an alpha value greater than 0.8, with a high value equal to 0.95. Therefore, it can be concluded that each component of the instrument was good and reliable.

**Table 5:** Cronbach’s alpha test for each component in the instrument.

Part	Component	Cronbach’s alpha	No of items
B	Knowledge about SOC	0.827	19
C	Success factors of SOC	0.858	17
D	Involvement of human, process and technology in SOC development and implementation	0.950	27
E	Evaluation of organizational cyber security monitoring	0.895	10

This study applied a quantitative method for data analysis. The data obtained from the questionnaires were analyzed and compiled according to the relevant components. The analysis was conducted using Statistical Package for Social Sciences (SPSS) for Windows version 25.0. There are four types of analyses performed: descriptive, normality, correlation, and regression.

The purpose of descriptive analysis is to obtain the percentage distribution and frequency of the mean value for all items in all parts. The percentage value was used to describe the results of the required components. At the same time, the mean value was calculated to measure the requirement and priority of constructing matrix grids for all items. A normality analysis was conducted to determine whether the data distribution was normal or otherwise. The results of the normality analysis are presented in Table 6. Based on the skewness and kurtosis values for all the parts, it can be concluded that it represents a normal distribution for all the data. This is because both values are situated between -2 and 2 for all the parts.

**Table 6:** Normality analysis test.

Part	Component	Skewness	Kurtosis
B	Knowledge about SOC	0.528	0.304
C	Success factors of SOC	-0.405	-0.330
D	Involvement of human, process and technology in SOC development and implementation	-0.194	-1.647
E	Evaluation of organizational cyber security monitoring	-0.614	-0.120

## Descriptive analysis

### General information of the respondents

**Fig 2:**
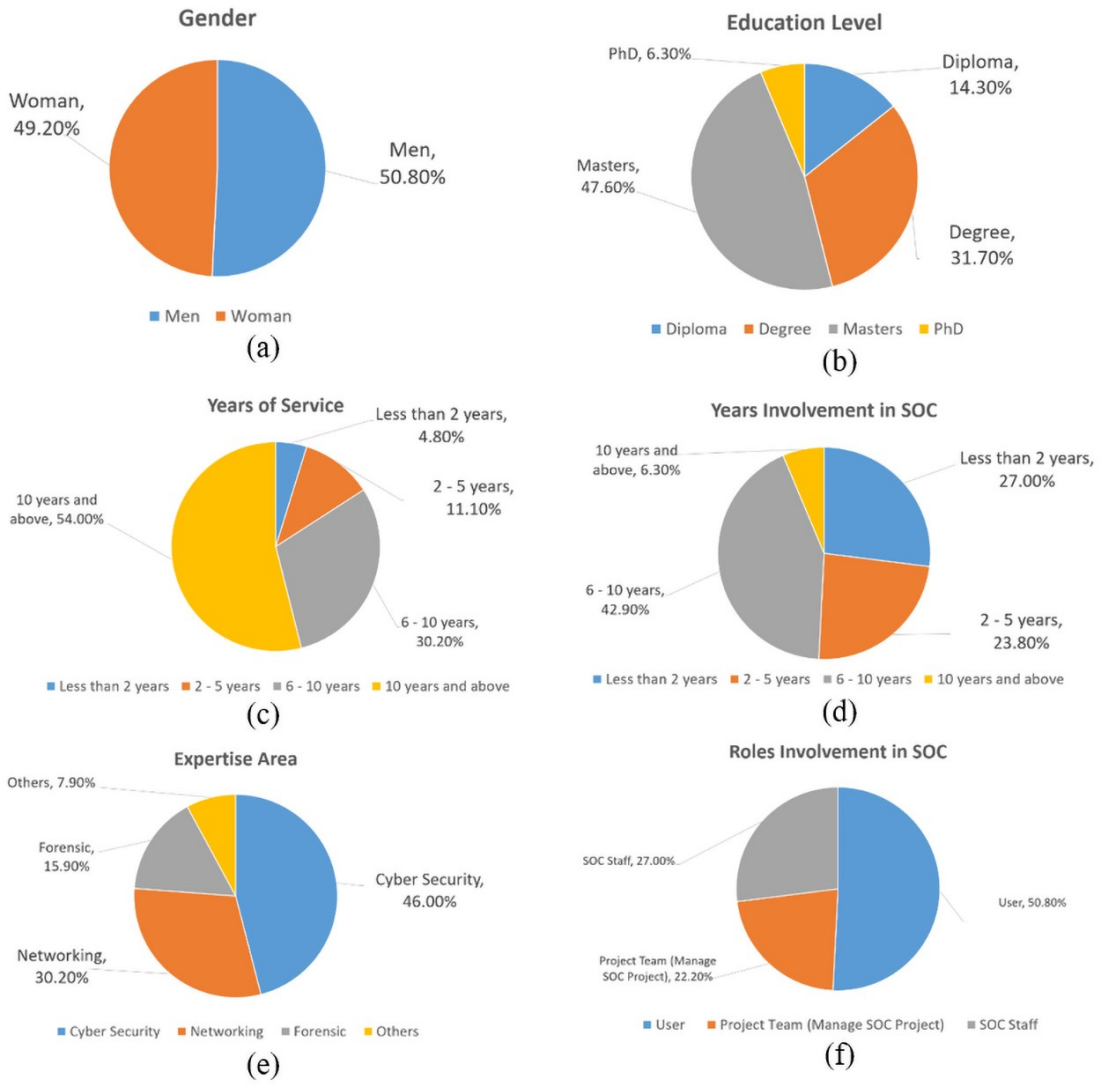
Compilation of general information of respondents. (a) gender group; (b) education level; (c) year of service in the agency; (d) experience with SOC; (e) expertise of the respondent; and (f) roles in SOC.

Descriptive analysis was used to obtain the distribution frequency and percentage of the respondents’ general information (Part A), knowledge about SOC (Part B), success of SOC (Part C), involvement of humans, processes, and technology in SOC (Part D), and evaluation of organizational cyber security monitoring (Part E). Fig 2 illustrates a compilation of the general information of the respondents.

According to Fig 2(a), in terms of gender, 32 respondents were male, while the rest were female. There was no significant difference between genders as male respondents exceeded only by one person. Regarding the level of education, nine of the respondents had a diploma, 20 respondents had a bachelor’s degree, 30 respondents had a master’s degree, and four persons had a Ph.D. Thus, for this group of respondents, the master has the highest number of respondents, and the Ph.D. is the lowest. By looking at the year of services, Fig 2(c) shows that three respondents served less than two years, seven respondents served between two to five years, and 54 respondents served for more than six years. This indicates that the majority of respondents have enough experience. Regarding involvement with the SOC, it can be seen that the majority of the respondents have engaged with SOC for a duration of 6 to 10 years. In addition, 6.3% have experienced in SOC for more than ten years.

Furthermore, based on the area of expertise, it appears that 46% or 29 respondents are involved in the management of cyber security, while the rest are in network management, forensic, and other specialties. Regarding the involvement role in SOC, the majority of the respondents were service recipients (users of the system), followed by the staff and project team of the SOC. In terms of professional certification, only 19 respondents provided feedback. Most respondents have certified ethical hacker (CEH) certification, while the rest have ISMS lead auditor certification.

### Evaluation on knowledge about SOC

The questionnaire in this part was intended to test respondents’ knowledge of SOC development and implementation. The questions were divided into four categories. The survey uses a nominal scale as measurement where the selection “Yes” is represented by 1, “No” represented by 2 and “Not Sure” represented by 3. Table 7 highlights the results of the frequency, percentage, mean, and standard deviation (SD) related to the questions about SOC. The overall mean value indicates that the respondents for the questionnaire were knowledgeable about SOC (M = 1.299, SD = 0.375). It can be classified as knowledgeable because this value is close to the actual selection value (e.g., “Yes” is equal to 1). From all the questions in this section, the high mean value is given by the question with the code SOC5 (M = 1.73, SD = 0.627), while the lowest mean is the code of SOC7 (M = 1.079, SD = 0.372). As such, this indicator indicates that most respondents knew the existing implementation of SOC. However, it can also be observed that most of the respondents do not know the status of SOC implementation in their organizations, as given in the SOC5 question. The respondents also expressed uncertainty regarding the question with the SOC3 code. This relates to the knowledge of the role and responsibilities of SOC.

**Table 7:** Questionnaire on general information about SOC.

Code	Statement	1	2	3	Mean	SD
SOC1	Do you know about SOC?	84.1%	6.3%	9.5%	1.254	0.621
SOC2	Do you know the reason for setting up SOC?	90.5%	4.8%	4.8%	1.142	0.470
SOC3	Do you know the role and responsibilities of SOC?	74.6%	9.5%	15.9%	1.412	0.754
SOC4	Do you know why SOC is established in the organization?	88.9%	4.8%	6.3%	1.174	0.524
SOC5	Does your organization implement SOC?	36.5%	54%	9.5%	1.73	0.627
SOC6	Has your organization monitored by any SOC?	77.8%	14.3%	7.9%	1.301	0.612
SOC7	Do you know any existing SOC?	95.2%	1.6%	3.2%	1.079	0.372
	**Overall Mean and SD**				1.299	0.375

Next, the questionnaire focuses on the respondents’ knowledge of the SOC framework. The overall findings of the SOC framework are listed in Table 8. Thus, it can be seen that overall, the respondents do not have basic knowledge of the SOC framework (M = 2.005, SD = 0.420). However, the distribution score for each question shows that the respondents did not know three out of the six questions. These questions are given by the codes SOC8, SOC9, and SOC12. These questions are related to knowledge of the SOC model, the standard for SOC establishment, and the differences between SOC in the country and overseas. This scenario might be associated with the demographics of the respondents, as most of them come from the user perspective. Hence, the respondents were not involved in SOC development, and knowledge from these three questions was essential. This argument is supported by observing the distribution of the respondents that score on the “Not Sure” category. The score in this category was slightly high (e.g., between 27% and 46% of the respondents). However, most respondents agree that academic and research literature can assist in the development and implementation of SOC. The majority also agreed that SOC implementation must be accorded to the organization’s objectives.

**Table 8:** Questionnaire on the framework of SOC.

Code	Statement	1	2	3	Mean	SD
SOC8	Do you know the type of model used to develop and implement the SOC?	25.4%	47.6%	27%	2.015	0.729
SOC9	Do you know the standards that apply to developing and implementing SOC?	22.2%	49.2%	28.6%	2.063	0.715
SOC10	Is the existing SOC framework working effectively and efficiently?	34.9%	19%	46%	2.111	0.9
SOC11	Is the SOC implemented by the organization meets the goals and objectives?	49.2%	4.8%	46%	1.968	0.983
SOC12	Do you know the difference between the SOC frameworks implemented locally and abroad?	9.5%	65.1%	25.4%	2.158	0.573
SOC13	Do academic and research literature assist in the development and implementation of SOC?	63.5%	1.6%	34.9%	1.714	0.957
	**Overall Mean and SD**				2.005	0.420

In respect of the knowledge of the function of SOC among the respondents, the overall mean value indicated that the respondents had little knowledge about the topic (M = 1.801, SD = 0.562). However, similar to the previous scenario with the framework of SOC, it seems that the distribution for respondents that score for “Not Sure” is still high, especially for three questions: SOC15, SOC16, and SOC17. In addition, it also highlights that most respondents do not know the basis for selecting the SOC function (e.g., SOC15). In contrast, the respondents knew the essential functions and minimum requirements of SOC. Further, the respondents strongly agreed that the current functions of the SOC could identify the latest cyberattacks. The distribution of respondents who agreed with these criteria was more than 50%. The overall findings on the knowledge of the functions of SOC among the respondents are highlighted in Table 9.

**Table 9:** Knowledge on the functions of SOC among the respondents.

Code	Statement	1	2	3	Mean	SD
SOC14	Do you know the minimum functions required for the SOC?	73%	17.5%	9.5%	1.365	0.655
SOC15	Do you know the basis for selecting the SOC functions during development?	28.6%	33.3%	38.1%	2.095	0.817
SOC16	Are the existing functions of SOC in line with current technology development?	38.1%	20.6%	41.3%	2.031	0.897
SOC17	Are the existing functions in SOC capable of identifying the latest cyberattacks?	58.7%	11.1%	30.2%	1.714	0.905
	**Overall Mean and SD**				1.801	0.562

Overall, the respondents were slightly aware of the general success factors for SOC (M = 1.531, SD = 0.552). Further, both survey questions (SOC18 and SOC19) have a high-frequency score for “Yes” as shown in Table 10. Nevertheless, the survey also indicates that the respondents agree that the success of the development and implementation of SOC depends on certain factors.

**Table 10:** Questionnaire on the general success factor for SOC.

Code	Statement	1	2	3	Mean	SD
SOC18	Do you know the factors that contribute to the success of SOC development and implementation?	42.9%	17.5%	39.7%	1.968	0.915
SOC19	Do you believe certain factors can contribute to the success of SOC development and implementation?	95.2%	0%	4.8%	1.095	0.429
	**Overall Mean and SD**				1.531	0.552

### Evaluation on success factors for SOC

As previously stated in Section 4, the questionnaire in this section is to test the respondents’ knowledge and point of view about the success factors in developing and implementing the SOC. It will involve a questionnaire based on a five-point Likert scale. The findings of the questionnaire are presented in Table 11. The results show that the overall mean value agrees with the SOC success factor (M = 4.416, SD = 0.313). Thus, the questionnaire shows that the respondents agreed with all the success factors of the SOC. As highlighted in Table 11, the top management support (e.g., FKS2) represents the highest mean value for the success factor of SOC (M = 4.888, SD = 0.316). This is also supported by the majority of the respondents who strongly agree on this factor (i.e., representing 88.9%). However, the question “Do you agree that not all factors should be implemented to determine the success of SOC development and implementation?” represents the lowest mean (M = 3.349, SD = 1.138).

**Table 11:** Questionnaire on ten success factors of SOC.

Code	Statement	1	2	3	4	5	Mean	SD
FKS1	Do you agree that several specific factors determine the success of the SOC?	0%	0%	6.3%	47.6%	46%	4.396	0.610
FKS2	Do you agree that the top management support factors can determine the success of SOC?	0%	0%	0%	11.1%	88.9%	4.888	0.316
FKS3	Do you agree that financial factors can determine the success of the SOC?	0%	1.6%	0%	20.6%	77.8%	4.746	0.537
FKS4	Do you agree that strategic factors determine the success of the SOC?	0%	0%	3.2%	27%	69.8%	4.666	0.538
FKS5	Do you agree that human factors can determine the success of the SOC?	0%	0%	0%	23.8%	76.2%	4.761	0.429
FKS6	Do you agree that the process factors can determine the success of SOC?	0%	0%	0%	22.2%	77.8%	4.777	0.419
FKS7	Do you agree that technology factors can determine the success of SOC?	0%	0%	7.9%	20.6%	71.4%	4.634	0.629
FKS8	Do you agree that environmental factors can determine the success of the SOC?	0%	0%	1.6%	61.9%	36.5%	4.349	0.513
FKS9	Do you agree that analysis and reporting can determine the success of SOC?	0%	0%	3.2%	52.4%	44.4%	4.412	0.557
FKS10	Do you agree that the physical space factor can determine the success of the SOC?	0%	1.6%	25.4%	54%	19%	3.904	0.711
FKS11	Do you agree that the improvement factors can determine the success of SOC?	0%	0%	1.6%	57.1%	41.3%	4.396	0.524
FKS12	Do you agree that all the above factors influence the success of the SOC?	0%	0%	0%	54%	46%	4.460	0.502
FKS13	Are there any of these factors that have been implemented in an organization to ensure the success of SOC?	0%	1.6%	23.8%	46%	28.6%	4.015	0.772
FKS14	Do you agree that all of the above factors should be implemented in determining the success of SOC?	0%	0%	27%	42.9%	30.2%	4.031	0.761
FKS15	Do you agree that not all of the above factors should be implemented to determine the success of SOC development and implementation?	7.9%	14.3%	27%	36.5%	14.3%	3.349	1.138
FKS16	Do you agree that humans, processes, and technologies are interrelated in determining the success of SOC?	0%	0%	0%	30.2%	69.8%	4.698	0.462
FKS17	Do you agree that humans, processes, and technologies are equally important in determining the success of SOC?	0%	0%	3.2%	34.9%	61.9%	4.587	0.557
	**Overall Mean and SD**						4.416	0.313

The second highest mean is the process or procedure (FKS6), with 77.8% respondents strongly agreeing with this factor. This is followed by the human factor (FKS5), where the score for strongly agree is 76.2%. Subsequently, the technology represents the least among the human-process-technology factors, as only 71.4% of the respondents strongly agreed that this factor is crucial for the development and implementation of SOC.

### Evaluation on the involvement of humans, processes, and technology in SOC development and implementation

Similarly, the measurement for this part of the questionnaire was based on a five-point Likert scale. Overall, the respondents agreed with the importance of human, process, and technology in developing and implementing SOC. From the observation, the overall mean value for human, process, and technology factors was above 4. The process factor led to the value of the overall mean (M = 4.539, SD = 0.487). Hence, it was selected as the most crucial factor affecting the success of the SOC. Then, it is followed by the human factor, with the value of the overall mean equal to 4.451, while the technology factor comes last with a value of 4.392. This finding is also in line with the results of the previous section.

The statements on the process factor focus on the need for established procedures and their documentation. The procedures must also be tallied with the existing functions in the SOC, such as monitoring, incident response, detection, and others. The detailed questionnaire found that more than 50% of the respondents strongly agreed with three statements (D2-MPT13, D2-MPT14 and D2-MPT15) as listed in Table 12. In addition, the mean value for all these statements is the same, which is equal to 4.539.

**Table 12:** Questionnaire results of process factors.

Code	Statement	1	2	3	4	5	Mean	SD
MPT13	Do you agree that processes and procedures need to be developed for each function performed by the SOC?	0%	0%	0%	46%	54%	4.539	0.502
MPT14	Do you agree that the developed processes and procedures need to be regularly documented?	0%	0%	1.6%	42.9%	55.6%	4.539	0.533
MPT15	Do you agree that the processes and procedures that are documented regularly can facilitate the task at SOC?	0%	0%	0%	46%	54%	4.539	0.502
	**Overall Mean and SD**						4.539	0.487

Table 13 outlines the findings of human factors in the development and implementation of SOC. It shows that the respondents agreed with all the statements on the human factor. The high mean value is in the general context of training, with a mean of 4.451 and a standard deviation of 0.475. Thus, this finding highlights that training is crucial in SOC, as it produces highly skilled and knowledgeable employees. On the other hand, the lowest mean was for forensic training for employees (M = 4.095, SD = 0.945). This finding shows that forensic training is a minor requirement for SOC compared to incident management, threat intelligence, and security monitoring. In addition, the respondents emphasized the importance of training in addressing the shortage of cyber security experts. This is shown in statement MPT12, where 69.8% of the respondents strongly agreed.

**Table 13:** Questionnaire results for the human factors.

Code	Statement	1	2	3	4	5	Mean	SD
MPT1	Do you agree that the officer in charge of the SOC should have the necessary knowledge and skills?	0%	0%	4.8%	34.9%	60.3%	4.555	0.589
MPT2	Do you agree that an officer on duty in the SOC should be aware in the field of security monitoring?	0%	0%	6.3%	44.4%	49.2%	4.428	0.614
MPT3	Do you agree that the officer in charge of the SOC needs to know in the field of threat intelligence?	0%	0%	12.7%	46%	41.3%	4.285	0.682
MPT4	Do you agree that an officer on duty in the SOC should be knowledgeable in the area of incident management?	0%	0%	4.8%	33.3%	61.9%	4.571	0.587
MPT5	Do you agree that an officer on duty in the SOC should be knowledgeable in the forensic field?	3.2%	1.6%	15.9%	41.3%	38.1%	4.095	0.945
MPT6	Do you agree that knowledge in the areas mentioned above is critical for a practitioner in charge of SOC?	0%	0%	6.3%	42.9%	50.8%	4.444	0.616
MPT7	Do you agree that there are other areas of knowledge and skills required for officers working in SOC?	0%	0%	12.7%	47.6%	39.7%	4.269	0.676
MPT8	Do you agree that effective communication is a necessary soft skill for the officer in charge of SOC?	0%	0%	20.6%	22.2%	57.2%	4.365	0.809
MPT9	Do you agree that the spirit of cooperation is a necessary soft skill for an officer in charge of SOC?	0%	0%	3.2%	36.5%	60.3%	4.571	0.559
MPT10	Do you agree that soft and technical skills are equally crucial for SOC officers?	0%	0%	3.2%	41.3%	55.5%	4.523	0.563
MPT11	Do you agree that training an officer in the SOC is vital to produce highly skilled and knowledgeable employees?	0%	0%	1.6%	30.2%	68.2%	4.666	0.508
MPT12	Do you agree that training is one way to address the shortage of experts in cyber security?	0%	0%	6.4%	23.8%	69.8%	4.634	0.603
	**Overall Mean and SD**						4.451	0.475

Subsequently, 68.3% of the respondents strongly agreed that training for SOC officers is essential for producing highly skilled employees. The findings also highlight that most respondents agree that soft skills are equally crucial as technical skills. All statements on soft skills received a strong agreement with the respondents for more than 50%.

For the technology factor, the focus of the questionnaire is toward the functions in the SOC. The statements outline whether these functions are required for the development and implementation of SOC. Such functions involve monitoring, log collection, analysis, cyber forensics, and responding to cyber security incidents that occur in the organization. Furthermore, financial issues are considered as one of the elements in the technology factor.

The findings from Table 14 indicate that the respondents agree on all the statements on technology factors. The range of the mean for the statements was between 4.079 and 4.539. The statement on the financial issue where the implementation of SOC functions depends on the organization’s financial ability represents the highest mean value. It is also noted that 69.8% of the respondents strongly agreed with this statement.

**Table 14:** Questionnaire results for technology factors.

Code	Statement	1	2	3	4	5	Mean	SD
MPT16	Do you agree that SOC needs to implement a necessary scope of work?	0%	0%	4.8%	44.4%	50.8%	4.460	0.590
MPT17	Do you agree that monitoring, analysis, and response are the scope of the necessary work for SOC?	0%	0%	4.8%	44.4%	50.8%	4.460	0.590
MPT18	Do you agree that these functions need to be implemented in the SOC?	0%	1.6%	4.8%	46%	47.6%	4.396	0.660
MPT19	Do you agree that function monitoring and log collection management should be performed by SOC?	0%	0%	6.3%	39.7%	54%	4.476	0.618
MPT20	Do you agree that function analysis management needs to be carried out by SOC?	0%	0%	0%	46%	54%	4.539	0.502
MPT21	Do you agree that the function of incident management and response should be implemented by SOC?	0%	0%	3.2%	47.6%	49.2%	4.428	0.665
MPT22	Do you agree that the function of forensic management needs to be implemented by SOC?	1.6%	6.4%	9.5%	46%	36.5%	4.095	0.928
MPT23	Do you agree that SOC must implement the functions of basic cyber security operations and management?	0%	4.8%	8%	39.6%	47.6%	4.301	0.815
MPT24	Do you agree that all of the above functions have been performed by SOC?	0%	4.8%	20.6%	36.5%	38.1%	4.079	0.885
MPT25	Do you agree that the SOC that performs all the scopes (monitoring, analysis, and response) can protect the organization from threats and cyberattacks?	0%	0%	4.8%	42.9%	52.3%	4.476	0.591
MPT26	By considering the evolution of cyberattacks, do you agree that the function of threat intelligence management is necessary for SOC?	0%	0%	3.2%	47.6%	49.2%	4.460	0.562
MPT27	Do you agree that implementing these functions in the SOC depends on the organization’s financial ability/constraint?	0%	0%	8%	30.1%	61.9%	4.539	0.643
	**Overall Mean and SD**						4.392	0.509

In contrast, the statement of implementation of forensic management has the lowest mean, with 25.8% of the respondents disagreeing with it. The analysis management was selected as the most crucial function as its mean value was equal to 4.539, and 54% of the respondents strongly agreed to it. The same among respondents also agreed on the function of log collection and monitoring management. Moreover, the threat of intelligence management is also exhibited as a crucial function for SOC, as approximately 49.2% of respondents strongly agree with it. However, through MPT24, it is noted that about a quarter of the respondents disagree that all functions have been performed by SOC. Thus, some of these functions may be handled by means other than a complete system set. The statement MPT24 (M = 4.079, SD = 0.885) also had the lowest mean value among the statements.

### Evaluation on organization’s cyber security monitoring

This part of the questionnaire concerns the organization’s knowledge of cyber security strategies. It consists of several cyber security strategies, such as protection implementation, defining the organization’s cyber vulnerabilities, phases of cyber security, and readiness. In the first part of the questionnaire, the statements cover the importance of the organization’s strategies to protect the information security infrastructure, either by technology or non-technology-based. In addition, it provides an overview of whether these strategies have been implemented in the respondents’ organization. The questionnaire then focuses on the knowledge of the general type of vulnerabilities that attackers can exploit. It aims to obtain the respondents’ agreement on whether these vulnerabilities are from technology, process, and humans. It also covers knowledge of readiness by highlighting the prevention, detection, and correction controls.

The findings of the questionnaire are presented in Table 15. The results indicate that the respondents agree with all the statements on this part of the questionnaire, as the overall mean value is greater than 4 (M = 4.403, SD = 0.553). The statement on the importance of implementing cyber security strategies to protect the IT infrastructure (PEN1) represents the highest mean value in this questionnaire (M = 4.571, SD = 0.665). This is also supported by the majority of respondents who strongly agree with the statement. The finding (PEN2) also shows a strong agreement among the respondents regarding implementing the cyber security strategy. However, the PEN3 statement highlights that around ten respondents disagree that their organizations are well equipped with cyber security strategies, either technology or non-technology-based.

**Table 15:** Questionnaire results on the evaluation of organizational cyber security monitoring.

Code	Statement	1	2	3	4	5	Mean	SD
PEN1	Do you agree that organizations need to set up cyber security strategies to protect their information security infrastructure?	0%	0%	9.5%	23.8%	66.7%	4.571	0.665
PEN2	Do you agree that cyber security strategies can be implemented either through technology or non-technology-based?	0%	0%	6.4%	38%	55.6%	4.492	0.618
PEN3	Do you agree that these strategies (technology or non-technology-based) have been implemented in your organization?	0%	4.8%	11.1%	46%	38.1%	4.174	0.813
PEN4	Do you agree that the vulnerabilities in an organization’s cyber security are due to human, process, and technology factors?	0%	0%	11.1%	41.3%	47.6%	4.365	0.679
PEN5	Do you agree that organizational vulnerabilities in human, process, and technology aspects can be exploited by hackers/cyber attackers?	0%	0%	4.8%	34.9%	60.3%	4.555	0.589
PEN6	Do you agree that organizational weaknesses, especially in the human aspect, are among the factors that contribute to cyberattacks?	0%	0%	14.3%	28.6%	57.1%	4.428	0.734
PEN7	Do you agree that organizations need to be prepared to deal with threats and cyberattacks by performing the appropriate response mechanism?	0%	0%	4.8%	41.3%	53.9%	4.492	0.592
PEN8	Do you agree that three security levels (prevention, detection, and correction) need to be done in the organization?	0%	0%	11.1%	34.9%	54%	4.428	0.688
PEN9	Do you agree that there are three security levels, namely prevention, detection, and correction, that have been implemented in your organization?	0%	0%	15.9%	47.6%	36.5%	4.206	0.699
PEN10	Do you agree that three security levels, namely prevention, detection, and correction, can protect the organization from threats and cyberattacks?	0%	3.2%	9.5%	39.7%	47.6%	4.317	0.779
	**Overall Mean and SD**						4.403	0.553

Around 47.6% of the respondents strongly agree that the cyber security vulnerabilities are due to human, process, and technology factors. At the same time, another 41.3% agreed with the same statement. Thus, with a high proportion of the respondents agreeing on this issue, it can be seen that the majority also reach an agreement that the attacker can exploit these vulnerabilities, especially from the human factor (refer to PEN5 and PEN6). Further, 54% of the respondents strongly agreed that the organization needs to prepare cyber security readiness (PEN7) to handle cyberattacks.

## Correlation and regression analysis

Correlation analysis was implemented to identify the strengths and assess the relationships between the components. This study implements Pearson correlation analysis [46]. Thus, a correlation test is conducted on the data related to the three success factors of SOC, namely human, process, and technology. The results of the tests are presented in Table 16.

**Table 16:** Correlation test between human, process, and technology factors.

	Human	Process	Technology
Human	1	0.715**	0.926**
Process	0.715**	1	0.696**
Technology	0.926**	0.696**	1

The test identified a significantly strong correlation and also relationship between human and process factors (r = 0.715, p = 0.000). In addition, human factors also have a strong correlation with technology (r = 0.926, p = 0.000). In terms of the correlation between the process and technology factors, their relationship was also significantly strong (r = 0.696, p = 0.000). Therefore, this finding is in line with previous research that has been discussed in Section 3.

The regression analysis specifically measures the impact of the studied elements based on the identified independent variables. This was applied to determine the most crucial element. The baseline validation analysis was performed using a regression test to form the model of development and implementation of SOC. The determination coefficient (R Square) measures the proportionality of the dependent variable variance against the mean score of the independent variable. The higher the value, the greater the explanatory power of the regression model, as it represents the contribution of all variables to the model. In contrast, the strength values of the relationships between the variables were demonstrated by using beta values.

In this study, the independent variables were represented by human, process, and technology factors. These independent variables were tested for the success of SOC development and implementation, which represented the dependent variables. Table 17 shows the overall relationship between the determinants of the success of SOC development and implementation of human, process, and technology factors. Based on the ANOVA tests in Table 18, the results of the analysis show a significant value for the success of SOC. In addition, based on Table 19, the multi-regression test indicates that the process (Beta = 0.282, p = 0.05) and technology (Beta = 0.431, p = 0.05) are significantly associated with the success of SOC. The value of R2 = 0.712 indicates that 71.2% of the change in the success of SOC is due to process and technology factors. Further, the results also show that the technology factor has the most significant impact on determining the success of SOC, followed by the process. The human factor, in turn, has little impact and is not significant in determining the success of SOC.

**Table 17:** Regression model.

Model	R	R Square	Adjusted R Square	Std. Error of Estimate
1	0.844	0.712	0.698	0.30424

**Table 18:** ANOVA table.

	Sum of Squares	df	Mean Square	F	Sig.
Regression	13.518	3	4.506	48.680	.000
Residual	5.461	59	0.093		
Total	18.979	62			

**Table 19:** Multi regression test.

	Unstandardized Coeff.		Standardized Coeff.	T	Sig.
	B	Std. Error	beta		
(Constant)	-0.135	0.392		-0.344	0.732
Human	0.230	0.223	0.197	1.033	0.306
Process	0.321	0.114	0.283	2.812	0.007
Technology	0.468	0.202	0.431	2.313	0.024

## Model for development and implementation of cyber security operations centre (SOC)

**Fig 3:**
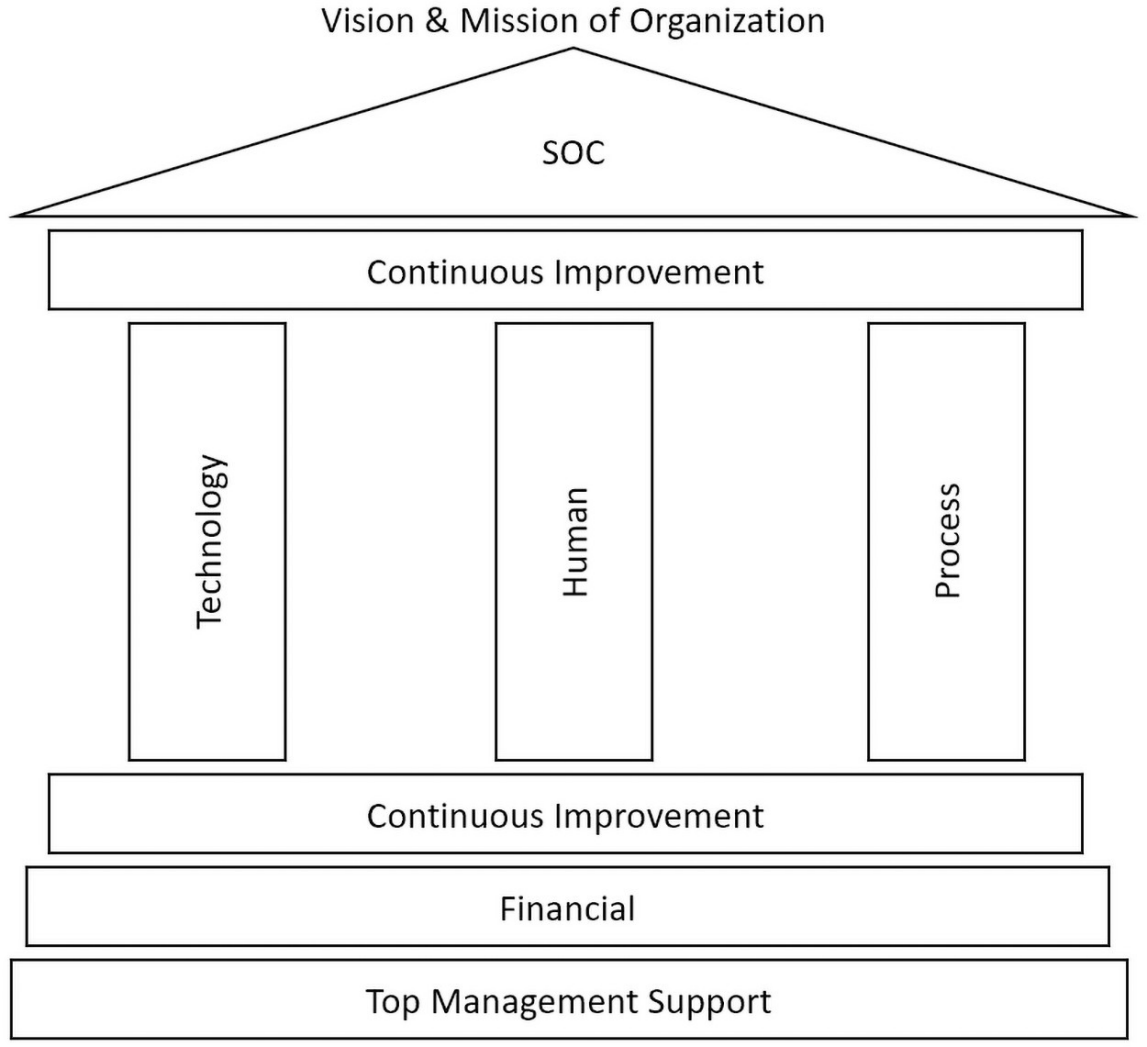
Model for the development and implementation of the SOC.

Fig 3 outlines the model for the successful development and implementation of SOC. It encompasses human, process, and technology factors. This model was developed based on a review of previous studies and the results of the descriptive analysis, correlation, and regression tests.

From the observation on 11 studies in Section 3, the conceptual model of the success factors of SOC was introduced, as discussed in Section 4. This conceptual model is shaped by humans, processes, and technology as the key factors. Further, prior studies also emphasize on continuous improvement as an essential factor for the success of SOC. Continuous improvement is considered vital because this factor ensures that the current SOC remains relevant over time. This factor must ensure that the SOC is up-to-date with recent trends in cyberattacks and technology. However, the financial factor is also considered because it plays a crucial role in ensuring that all other factors can be implemented. The development and implementation of SOC is closely linked to the financial ability of the organization.

Based on the descriptive analysis of the success factor of SOC, it was found that the top management support factor had the highest mean value (M = 4.888, SD = 0.316). This is supported by the fact that 88.9% of the respondents strongly agreed that this factor was the most influential factor in the development and implementation of SOC. Top management support is crucial in governance, where it can set clear directions, set priorities, and formulate long-term strategies for SOC implementation. The second factor that received the majority of respondents’ consent (77.8%) was the financial factor (M = 4.746, SD = 0.537). Therefore, by referring to the descriptive analysis as a whole, the top management, financial, human, process, and technology represent the key factors for the success of SOC, especially from respondents’ views and opinions. The correlation analysis, on the other hand, highlights that human, process, and technology factors have a strong and significant relationship with the success of SOC. Further, a 71.2% change in the success of SOC is due to the process and technology factors, as highlighted in the regression test.

SOC aims to protect the organization’s critical services by curbing and eradicating threats and cyberattacks. Its role supports the vision and mission of the organization to ensure that vital services can be smoothly implemented. Accordingly, the shape of this model represents the visual structure of SOC. It consists of a solid foundation and a central component in a vertical column, standing on the base, and supporting the roof.

Top management support and finances are defined as external factors that directly impact the success of the development and implementation of SOC. In this regard, these two factors are at the base layer to support the critical components of SOC, namely human, process, and technology. The top management support is located at the lowest base because this factor is described as the source of authority for the organization. Thus, the development and implementation of SOC can be supported through the direction and mandate from top management. Upon approval from the top management, financial allocation can be utilised to support the success of the SOC.

The vertical columns represent human, process, and technology factors. From the regression test results, it was found that humans are the least significant in human-process-technology factors in supporting the success of SOC. Hence, the human factor is placed in the center between the technology and process. In the absence of a human factor, the SOC structure (roof) remains strong as the support comes from technology and process. The last component that forms the SOC model is the continuous improvement factor. It is placed above and below the main components (human, process, and technology) to highlight that they are continually improving to ensure that the SOC remains relevant to the recent cyber security development. The details of the basic requirements of the crucial components of SOC identified in this study are shown in Table 20.

**Table 20:** Basic requirements for humans, processes, and technology.

Factor	Basic Requirements
Human	Training *Technical skills: Security monitoring Threat intelligence Incident management Forensic Soft skills: Communication Teamwork *Depend on SOC functionality
Process	Define processes and procedures for all functions in SOC Document the processes and procedures Smooth and consistent operation
Technology & Elements	Management of log monitoring and collection Analysis management Incident management and response Threat intelligence management Forensic management

## Conclusion

Based on prior studies and the analysis of the questionnaire on knowledge of SOC, this study proposes a model for the successful development and implementation of SOC. It is based on the critical factors of SOC, namely human, process, and technology, with support from external factors such as top management support, financial, and continuous improvement. For future work, it is suggested that the study conducts a combination of qualitative and quantitative methods to enhance the validity of the data. Further, in-depth research on each contributing factor will also be considered in future studies.

## Supporting information

S1 Appendix Profile of cyber security expert reviews for instruments evaluation. (PDF)

S1 File Data from questionnaire exercises. (XLSX)
