# Supplementary material for: Model for successful development and implementation of Cyber Security Operations Centre (SOC)
Source: PLoS One. 2021 Nov 19;16(11):e0260157. doi: 10.1371/journal.pone.0260157 (PMC8604312; doi:10.1371/journal.pone.0260157)
Supplement: S1 Appendix — (PDF) [file pone.0260157.s001.pdf]

Profile of Cybersecurity Expert Reviews for Instruments Evaluation

Expert	Position and Organization	Eligibility and Experience	Professional Certification
A	Senior Assistant Director, Agency A	▪ Ten years experience in cybersecurity<br>▪ Five years experience as Head of Malaysia Government Security Operational Centre project (MyGSOC)	▪ *ISO/IEC 27001 Lead Auditor*
B	Head of Digital Forensic Department, Agency B	▪ *Professional Technologist* (TS)<br>▪ Has extensive experience in handling computer crimes, forensic examination of computer crimes related to various law enforcement agencies in Malaysia	▪ *ISC2 ISLA 2011 Managerial Professional*<br>▪ *ASCLD/LAB certified Assessor*
C	*Specialist*, Agency B	▪ *Cybersecurity Specialist*<br>▪ Experience in cybersecurity especially in the web security application	▪ *Certified Ethical Hacker* (CEH)<br>▪ *EC-Council Certified Secure Programmer*<br>▪ *SANS-GWAPT* (*Web Application Penetration Testing*)
D	Senior Assistant Director, Agency A	▪ 15years experience in cybersecurity<br>▪ Project team leader for Government Public Key Infrastructure (GPKI)	▪ *PRINCE2®*<br>▪ *IT Disaster Recovery Implementer*<br>▪ *Disaster Recovery Professional (EC-DRP)*<br>▪ *ISO/IEC 27001 Lead Auditor*
E	*Associate Professor,* University A	▪ Chairman for Cybersecurity Research Centre in university<br>▪ Experience in artificial intelligence, pattern recognition, and computer vision	-

Profile of Cybersecurity Expert Reviews for Model Evaluation

Expert	Position and Organization	Eligibility and Experience	Professional Certification
**Expert I**	Deputy Director, Agency A	▪ 18 years experience in cybersecurity<br>▪ Hold a Professional Technologist (TS)<br>▪ Holds a Doctorate (Ph.D.) from Universiti Putra Malaysia in Computer Security<br>▪ Project Manager for Implementation of Digital Forensic in Public Sector<br>▪ Project Manager for Government Public Key Infrastructure	▪ *Certified Ethical Hacker* (CEH)<br>▪ *ISMS Lead Auditor*
**Expert II**	Chief Assistant Director, Agency A	▪ 15 years experience in cybersecurity<br>▪ Holds a Professional Technologist (TS)<br>▪ Holds a Master degree in Computer Science from Universiti Putra Malaysia: project on Modelling of Post-Incident Root Cause Analysis for Cross-Site Request Forgery (XSRF) Attack<br>▪ Responsible for providing cybersecurity consultation to public sector and government of Malaysia<br>▪ Manage incident response for public sector agencies	▪ *Certified Ethical Hacker* (CEH)<br>▪ *Computer Hacking Forensic Investigator* (CHFI)<br>▪ *EC-Council Certified Secure Programmer* (ECSP)<br>▪ *EC-Council Certified Security Analyst* (ECSA)<br>▪ *EC-Council Certified Incident Handler* (ECIH)

Example of Expert Review Evaluation on the Instruments of the Questionnaire

Note: the evaluation is conducted in the Malay language.

1.0 LATAR BELAKANG KAJI SELIDIK

Bahagian ini akan menerangkan mengenai ringkasan kajian, tujuan dan skop kaji selidik serta sumber kaji selidik.

1.1 Ringkasan Kajian

Kajian dilaksanakan berdasarkan penyataan masalah yang telah dikenal pasti hasil daripada kajian kesusasteraan yang dijalankan. Berdasarkan kajian kesusasteraan, serangan siber semakin berleluasa saban hari di seluruh dunia termasuk Malaysia. Kajian ke atas beberapa kes serangan siber menunjukkan peningkatan kemahiran penggodam dan teknik yang digunakan dalam mengeksploitasi kelemahan yang ada dalam manusia, proses dan teknologi. Evolusi serangan siber ini memberikan impak yang buruk kepada organisasi mahupun individu. Kajian ini akan memfokuskan kepada langkah balas yang boleh dilaksanakan oleh organisasi dan penyelesaian popular yang dikenal pasti adalah pelaksanaan Pusat Operasi Keselamatan Siber. Pusat Operasi Keselamatan Siber berupaya membantu organisasi untuk membendung dan membasmi insiden siber yang berlaku. Berdasarkan faktor kejayaan bagi pelaksanaan Pusat Operasi Keselamatan Siber, aspek manusia, proses dan teknologi adalah faktor yang mempunyai peratusan yang tertinggi dalam kajian lampau. Sehubungan itu, kajian ini akan memfokuskan kepada faktor kejayaan pembangunan dan pelaksanaan Pusat Operasi Keselamatan Siber di persekitaran sektor awam Malaysia bagi aspek manusia, proses dan teknologi. Kajian ini akan menggunakan pendekatan kajian secara kuantitatif bagi pengumpulan data dan analisis.

1.2 Objektif Dan Skop Kaji Selidik

Mendapatkan perspektif dan pandangan responden yang pernah bertugas atau terlibat dengan pelaksanaan Pusat Operasi Keselamatan Siber. Responden adalah terdiri daripada pegawai yang berkhidmat di sektor awam dan juga sektor swasta. Pandangan responden diperlukan bagi tujuan mengenal pasti faktor kejayaan pembangunan dan pelaksanaan Pusat Operasi Keselamatan Siber bagi persekitaran sektor awam Malaysia.

1.3 Sumber Kaji Selidik

Kaji selidik ini dihasilkan daripada kajian kesusasteraan yang dilaksanakan. Sumber kajian kesusasteraan ini terdiri daripada artikel jurnal, prosiding persidangan, laporan teknikal, laporan akhbar dan laporan statistik daripada laman sesawang daripada tahun 2011 sehingga tahun 2018.

2.0 SOALAN KAJI SELIDIK UNTUK ULASAN PAKAR

Bahagian ini akan menyenaraikan soalan yang akan dimasukkan ke dalam kaji selidik kajian kepada responden terlibat. Ulasan dan persetujuan pakar diperlukan bagi menentukan kerelevanan dan kepentingan soalan yang digunakan dalam kaji selidik kajian ini. Bentuk soalan yang diajukan kepada responden adalah secara berstruktur (tertutup) dan berpandu di mana jawapan tepat atau paling hampir kepada kenyataan atau soalan yang ditanya dipilih oleh responden. Kaji selidik juga menggunakan kaedah Skala Likert dengan pengukuran satu (1) hingga lima (5). **Soalan kaji selidik sebanyak 79 soalan akan terbahagi kepada lima (5) bahagian soalan seperti berikut:**

**Bil.**	**Komponen**	**Bahagian Soalan**
1	Maklumat Umum	Bahagian A
2	Pengetahuan Mengenai Pusat Operasi Keselamatan Siber	Bahagian B
3	Faktor Kejayaan Pusat Operasi Keselamatan Siber	Bahagian C
4	Penglibatan Faktor Manusia, Proses Dan Teknologi Dalam Pelaksanaan Dan Pembangunan Pusat Operasi Keselamatan Siber	Bahagian D
5	Penilaian Terhadap Pemantauan Keselamatan Siber Organisasi	Bahagian E

Pakar juga dimohon untuk menilai **kepentingan soalan melalui Pemberat** yang dinyatakan dalam jadual di bawah. Nilai pemberat yang diberikan oleh Pakar akan menentukan tahap kepentingan soalan yang dikemukakan. Pada masa yang sama, dapat memberikan maklumat tentang tahap kesediaan Pusat Operasi Keselamatan Siber di persekitaran sektor awam Malaysia.

**Definisi**	**Pemberat**
Amat Penting	4
Penting	3
Kurang Penting	2
Abaikan	1

BAHAGIAN A : MAKLUMAT UMUM

Soalan bahagian ini bertujuan untuk mendapatkan maklumat umum responden dan juga organisasi responden. Item-item yang terkandung dalam bahagian ini terdiri daripada **tujuh (7) soalan** yang diberi pilihan jawapan dan isi tempat kosong.

**Bah.**	**Kod Soalan**	**Soalan**	**ULASAN PAKAR**		
			**Tandakan √ di ruang berkaitan**		**Kepetingan Soalan**
			**Relevan**	**Tidak Relevan**	**Pemberat (4/3/2/1)**
A	A-MU1	Jantina	✓		3
	A-MU2	Taraf Pendidikan	✓		4
	A-MU3	Nama Agensi	✓		4
	A-MU4	Tempoh Berkhidmat	✓		4
	A-MU5	Bidang Kepakaran	✓		4
	A-MU6	Pensijilan Berkaitan	✓		4
	A-MU7	Tahap Penglibatan Dalam Pusat Operasi Keselamatan Siber (Secara Langsung / Tidak Langsung)	✓		3

ULASAN PAKAR: BAHAGIAN A

Sila tandakan X bagi ruang yang di kehendaki.

X	Bersetuju
	Tidak bersetuju

Sekiranya tidak bersetuju, sila nyatakan kod soalan dan kenapa tidak bersetuju:

Catatan dan Cadangan Penambahbaikan (sekiranya ada):

1) Tempoh berkhidmat dalam Pusat Operasi Keselamatan Siber.

2) Pensijilan professional berkaitan

BAHAGIAN B : PENGETAHUAN MENGENAI PUSAT OPERASI KESELAMATAN SIBER

Soalan bahagian ini bertujuan untuk menguji pengetahuan responden mengenai pelaksanaan dan pembangunan Pusat Operasi Keselamatan Siber. Item-item yang terkandung dalam bahagian ini terdiri daripada **17 soalan** yang diukur menggunakan skala pengukuran nominal dengan pemilihan "Ya", "Tidak" dan "Tidak Pasti" sebagai pilihan jawapan.

Bah.	Kod Soalan	Soalan	ULASAN PAKAR		
			Tandakan √ di ruang berkaitan		Kepentingan Soalan
			Relevan	Tidak Relevan	Pemberat (4/3/2/1)
**B1**	**Maklumat Umum Mengenai Pusat Operasi Keselamatan Siber**				
	B1-SOC1	Adakah anda tahu apakah Pusat Operasi Keselamatan Siber?	✓		4
	B1-SOC2	Adakah anda tahu kenapakah perlu wujudkan Pusat Operasi Keselamatan Siber?	✓		4
	B1-SOC3	Adakah anda tahu apakah peranan Pusat Operasi Keselamatan Siber?	✓		4
	B1-SOC4	Adakah anda tahu apakah tanggungjawab Pusat Operasi Keselamatan Siber?	✓		4
	B1-SOC5	Adakah anda tahu mengapakah organisasi memerlukan perkhidmatan Pusat Operasi Keselamatan Siber?	✓		4
**Sumber**		(Arimatsu et al. 2018; Ernst & Young 2014; IBM Global Technology Services 2013; Kowtha et al. 2012; Onwubiko 2015; RSA Technical Brief 2014; Schinagl et al. 2015; Torress 2015)			
**B2**	**Rangka Kerja Pusat Operasi Keselamatan Siber**				
	B2-SOC6	Adakah anda tahu apakah model yang digunakan untuk membangunkan dan melaksanakan Pusat Operasi Keselamatan Siber?	✓		3
	B2-SOC7	Adakah anda tahu apakah standard yang digunakan untuk membangunkan dan melaksanakan Pusat Operasi Keselamatan Siber?	✓		4
	B2-SOC8	Adakah rangka kerja Pusat Operasi Keselamatan Siber yang sedia ada efektif dan berkesan?	✓		3
	B2-SOC9	Adakah Pusat Operasi Keselamatan Siber dilaksanakan selaras dengan objektif dan halatuju yang telah ditetapkan oleh organisasi?	✓		3
			✓		3
			✓		4

Bah.	Kod Soalan	Soalan	ULASAN PAKAR: Tandakan √ di ruang berkaitan		Kepetingan Soalan
			Relevan	Tidak Relevan	Pemberat (4/3/2/1)
	B2-SOC10	Adakah anda tahu perbezaan rangka kerja Pusat Operasi Keselamatan Siber dalam dan luar negara?			
	B2-SOC11	Adakah kajian kesusasteraan akademik membantu dalam merangka pembangunan dan pelaksanaan Pusat Operasi Keselamatan Siber?			
**Sumber**		(MDEC 2017; Onwubiko 2015; Schinagl et al. 2015)			
**B3**	**Fungsi Pusat Operasi Keselamatan Siber**				
	B3-SOC12	Adakah anda tahu fungsi asas/minimum yang perlu ada di Pusat Operasi Keselamatan Siber?	✓		3
	B3-SOC13	Adakah anda tahu apakah fak tor yang mempengaruhi pemilihan fungsi Pusat Operasi Keselamatan Siber?		✓	3
	B3-SOC14	Adakah fungsi sedia ada yang dijalankan di Pusat Operasi Keselamatan Siber kini mengikut perkembangan teknologi semasa?	✓		3
	B3-SOC15	Adakah fungsi sedia ada di Pusat Operasi Keselamatan Siber berupaya untuk mengenal pasti serangan siber yang terkini?	✓		4
**Sumber**		(Arimatsu et al. 2018; Jacobs et al. 2013; Onwubiko 2015; Schinagl et al. 2015)			
**B4**	**Faktor Kejayaan Pusat Operasi Keselamatan Siber**				
	B4-SOC16	Adakah anda tahu apakah faktor yang menyumbang kepada kejayaan pelaksanaan dan pembangunan Pusat Operasi Keselamatan Siber?	✓		4
	B4-SOC17	Adakah anda percaya faktor tertentu boleh menyumbang kepada kejayaan pelaksanaan dan pembangunan Pu sat Operasi Keselamatan Siber?	✓		3
**Sumber**		(Ernst & Young 2014; IBM Global Technology Services 2013; Mansfield-Devine 2016; MDEC 2017; Onwubiko 2015; RSA Technical Brief 2014; Schinagl et al. 2015; Sundaramurthy et al. 2017; Torress 2015)			

ULASAN PAKAR: BAHAGIAN B

Sila tandakan X bagi ruang yang di kehendaki.

[X] Bersetuju

[ ] Tidak bersetuju

Sekiranya tida k bersetuju, sila nyatakan kod soalan dan kenapa tidak bersetuju:

____

____

Catatan dan Cadangan Penambahbaikan (sekiranya ada):

- Perlu tahu maklumat dan data apa yang akan di pantau dan analisis serta saiznya.
- Pekabentuk dan senibina SOC juga perlu dimasukkan dlm survey ini.

faktor kejayaan pelaksanaan dan pembangunan Pusat Operasi Keselamatan Siber. Item-item yang terkandung dalam bahagian ini terdiri daripada **17 soalan** yang diukur menggunakan skala likert dengan pemilihan "Sangat Setuju", "Setuju", "Sederhana Setuju", "Tidak Setuju" dan "Sangat Tidak Setuju" sebagai pilihan jawapan.

**Bah.**	**Kod Soalan**	**Soalan**	**ULASAN PAKAR**		
			**Tandakan √ di ruang berkaitan**		**Kepetingan Soalan**
			**Relevan**	**Tidak Relevan**	**Pemberat (4/3/2/1)**
C	C-FKS1	Adakah anda bersetuju bahawa terdapat beberapa faktor tertentu yang menentukan kejayaan pelaksanaan dan pembangunan Pusat Operasi Keselamatan Siber?	✓		3
	C-FKS2	Adakah anda bersetuju faktor Sokongan Pengurusan Atasan boleh menentukan kejayaan pelaksanaan dan pembangunan Pusat Operasi Keselamatan Siber?	✓		4
	C-FKS3	Adakah anda bersetuju faktor Kewangan boleh menentukan kejayaan pelaksanaan dan pembangunan Pusat Operasi Keselamatan Siber?	✓		4
	C-FKS4	Adakah anda bersetuju faktor Strategi boleh menentukan kejayaan pelaksanaan dan p embangunan Pusat Operasi Keselamatan Siber?	✓		4
	C-FKS5	Adakah anda bersetuju faktor Manusia boleh menentukan kejayaan pelaksanaan dan pembangunan Pusat Operasi Keselamatan Siber?	✓		4
	C-FKS6	Adakah anda bersetuju faktor Proses boleh menentukan kejayaan pelaksanaan dan pembangunan Pusat Operasi Keselamatan Siber?	✓		4
	C-FKS7	Adakah anda bersetuju faktor Teknologi boleh menentukan kejayaan pelaksanaan dan pembangunan Pusat Operasi Keselamatan Siber?	✓		4
	C-FKS8	Adakah anda bersetuju faktor *Persekitaran boleh menentukan kejayaan pelaksanaan dan	✓		3

Bah.	Kod Soalan	Soalan	ULASAN PAKAR		
			Tandakan √ di ruang berkaitan		Kepetingan Soalan
			Relevan	Tidak Relevan	Pemberat (4/3/2/1)
		pembangunan Pusat Operasi Keselamatan Siber?<br><br>**Kakitangan Pusat Operasi Keselamatan Siber perlu mempunyai pengetahuan mengenai keutamaan dan hala tuju organisasi bagi memastikan tindak balas yang diambil adalah yang paling tepat dan sesuai.*			
	C-FKS9	Adakah anda bersetuju faktor *Analisis dan Laporan boleh menentukan kejayaan pelaksanaan dan pembangunan Pusat Operasi Keselamatan Siber?<br><br>**Pusat Operasi Keselamatan Siber perlu berupaya untuk melaksanakan analisis data ke atas pelbagai sistem dan peralatan untuk mengeluarkan laporan yang le ngkap dan komprehensif.*	✓		4
	C-FKS10	Adakah anda bersetuju faktor *Ruang Fizikal boleh menentukan kejayaan pelaksanaan dan pembangunan Pusat Operasi Keselamatan Siber?<br><br>**Pusat Operasi Keselamatan Siber perlu ditempatkan di kawasan yang selamat dan mempunyai kemudahan yang lengkap. Mewujudkan lokasi yang khusus boleh memendekkan masa tindak balas ke atas sesuatu insiden dan juga menggalakkan perkongsian pengetahuan serta semangat kerja berpasukan.*	✓		4
	C-FKS11	Adakah anda bersetuju faktor Penambahbaikan Berterusan boleh menentukan kejayaan pelaksanaan dan pembangunan Pusat Operasi Keselamatan Siber?	✓		4
	C-FKS12	Adakah anda bersetuju terdapat antara faktor-faktor ini yang mempengaruhi kejayaan pelaksanaan dan pembangunan Pusat Operasi Keselamatan Siber?	✓		3
	C-FKS13	Adakah terdapat faktor-faktor ini yang telah dilaksanakan di organisasi anda bagi memastikan kejayaan kejayaan	✓		4
			✓		4

Bah.	Kod Soalan	Soalan	ULASAN PAKAR		
			Tandakan √ di ruang berkaitan		Kepetingan Soalan
			Relevan	Tidak Relevan	Pemberat (4/3/2/1)
		pelaksanaan dan pembangunan Pusat Operasi Keselamatan Siber?			
	C-FKS14	Adakah anda bersetuju bahawa kesemua faktor di atas perlu dilaksanakan dalam menentukan kejayaan pelaksanaan dan pembangunan Pusat Operasi Keselamatan Siber?			
	C-FKS15	Adakah anda bersetuju bahawa hanya beberapa faktor utama sahaja perlu dilaksanakan dalam menentukan kejayaan pelaksanaan dan pembangunan Pusat Operasi Keselamatan Siber?		√	3
	C-FKS16	Adakah anda bersetuju bahawa terdapat kaitan antara faktor manusia, proses dan teknologi dalam menentukan kejayaan pelaksanaan dan pembangunan Pusat Operasi Keselamatan Siber?	√		4
	C-FKS17	Adakah anda bersetuju bahawa faktor manusia, proses dan teknologi adalah sama-sama penting dalam menentukan kejayaan pelaksanaan dan pembangunan Pusat Operasi Keselamatan Siber?	√		4
Sumber		(Ernst & Young 2014; IBM Global Technology Services 2013; Mansfield-Devine 2016; MDEC 2017; Onwubiko 2015; RSA Technical Brief 2014; Schinagl et al. 2015; Sundaramurthy et al. 2017; Torress 2015)			

ULASAN PAKAR: BAHAGIAN C

Sila tandakan X bagi ruang yang di kehendaki.

- [X] Bersetuju
- [ ] Tidak bersetuju

Sekiranya tidak bersetuju, sila nyatakan kod soalan dan kenapa tidak bersetuju:

C-FKS15: Bahasa soalan ini adalah leading question kerana menggunakan perkataan 'utama'.

Catatan dan Cadangan Penambahbaikan (sekiranya ada):

C-FKS15: Adakah anda setuju bahawa tidak perlu melaksanakan semua faktor di atas dalam menentukan...

BAHAGIAN D : PENGLIBATAN FAKTOR MANUSIA, PROSES DAN TEKNOLOGI DALAM PELAKSANAAN DAN PEMBANGUNAN PUSAT OPERASI KESELAMATAN SIBER

Soalan bahagian ini bertujuan untuk menguji pengetahuan dan mendapakan pandangan responden mengenai penglibatan faktor manusia, proses dan teknologi dalam pelaksanaan dan pembangunan Pusat Operasi Keselamatan Siber. Item-item yang terkandung dalam bahagian ini terdiri daripada **28 soalan** yang diukur menggunakan skala likert dengan pemilihan "Sangat Setuju", "Setuju", "Sederhana Setuju", "Tidak Setuju" dan "Sangat Tidak Setuju" sebagai pilihan jawapan.

Bil.	Kod Soalan	Soalan	ULASAN PAKAR		
			Tandakan √ di ruang berkaitan		Kepetingan Soalan
			Relevan	Tidak Relevan	Pemberat (4/3/2/1)
**D1**	**Faktor Manusia**				
	D1-MPT1	Adakah anda bersetuju pegawai yang bertugas di Pusat Operasi Keselamatan Siber perlu mempunyai kemahiran teknikal?	✓		4
	D1-MPT2	Adakah anda bersetuju pegawai yang bertugas di Pusat Operasi Keselamatan Siber perlu mempunyai p engetahuan teknikal?	✓		4
	D1-MPT3	Adakah anda bersetuju pegawai yang bertugas di Pusat Operasi Keselamatan Siber perlu mempunyai pengetahuan dalam bidang *Pemantauan Keselamatan?<br><br>**Security Monitoring*	✓		4
	D1-MPT4	Adakah anda bersetuju pegawai yang bertugas di Pusat Operasi Keselamatan Siber perlu mempunyai pengetahuan dalam bidang Kepintar an Ancaman?<br><br>**Threat Intelligence*	✓		4
	D1-MPT5	Adakah anda bersetuju pegawai yang bertugas di Pusat Operasi Keselamatan Siber perlu mempunyai pengetahuan dalam bidang Pengurusan Insiden?	✓		4
	D1-MPT6	Adakah anda bersetuju pegawai yang bertugas di Pusat Operasi Keselamatan Siber perlu mempunyai pengetahuan dalam bidang Forensik?	✓		4
	D1-MPT7	Adakah anda bersetuju pengetahuan dalam bidang yang dinyatakan di atas adalah sangat penting bagi pegawai yang bertugas di Pusat Operasi Keselamatan Siber?	✓		3

Bil.	Kod Soalan	Soalan	ULASAN PAKAR		
			Tandakan √ di ruang berkaitan		Kepetingan Soalan
			Relevan	Tidak Relevan	Pemberat (4/3/2/1)
	D1-MPT8	Adakah anda bersetuju terdapat bidang pengetahuan dan kemahiran lain yang perlu bagi pegawai yang bertugas di Pusat Operasi Keselamatan Siber?	✓		3
**Sumber**		(Arimatsu et al. 2018; IBM Global Technology Services 2013; Mansfield-Devine 2016; McAfee & Intel Security 2016; Onwubiko 2015; Sundaramurthy et al. 2014; Torress 2015)			
	D1-MPT9	Adakah anda bersetuju *kemahiran insaniah seperti komunikasi berkesan perlu bagi pegawai yang bertugas di Pusat Operasi Keselamatan Siber?<br><br>**Soft Skills*	✓		4
	D1-MPT10	Adakah anda bersetuju kemahiran insaniah seperti semangat kerjasama perlu bagi pegawai yang bertugas di Pusat Operasi Keselamatan Siber?	✓		4
	D1-MPT11	Adakah anda bersetuju kemahiran insaniah dan kemahiran teknikal adalah sama-sama penting bagi pegawai yang bertugas di Pusat Operasi Keselamatan Siber?	✓		3
**Sumber**		(Arimatsu et al. 2018; RSA Technical Brief 2014; Sundaramurthy et al. 2017)			
	D1-MPT12	Adakah anda bersetuju bahawa latihan bagi pegawai yang bertugas di Pusat Operasi Keselamatan Siber sangat penting bagi melahirkan pegawai yang berkemahiran tinggi dan berpenget ahuan?	✓		4
	D1-MPT13	Adakah anda bersetuju bahawa latihan adalah salah satu cara untuk mengatasi kekurangan tenaga pakar dalam bidang keselamatan siber?	✓		4
**Sumber**		(McAfee & Intel Security 2016; Onwubiko 2015; Sundaramurthy et al. 2014; Torress 2015)			
**D2**	**Faktor Proses**				
	D2-MPT14	Adakah anda bersetuju proses perlu dibangunkan bagi setiap fungsi yang dijalankan oleh Pusat Operasi Keselamatan Siber?	✓		4
	D2-MPT15	Adakah anda bersetuju proses yang dibangunkan perlu didokumentasi secara teratur?	✓		4
	D2-MPT16	Adakah anda bersetuju proses yang didokumentasi secara teratur dapat memudahkan proses kerja di Pusat Operasi Keselamatan Siber?	✓		4
**Sumber**		(Ernst & Young 2014; McAfee & Intel Security 2016; RSA Technical Brief 2014; Schinagl et al. 2015; Torress 2015)			

Bil.	Kod Soalan	Soalan	ULASAN PAKAR		
			Tandakan √ di ruang berkaitan		Kepetingan Soalan
			Relevan	Tidak Relevan	Pemberat (4/3/2/1)
**D3**	**Faktor Teknologi**				
	D3-MPT17	Adakah anda bersetuju terdapat beberapa skop kerja asas yang perlu dilaksanakan oleh Pusat Operasi Keselamatan Siber?	✓		4
	D3-MPT18	Adakah anda berset uju bahawa skop Pemantauan, Analisis dan Tindak balas adalah skop kerja utama yang perlu dilaksanakan oleh Pusat Operasi Keselamatan Siber?	✓		4
	D3-MPT19	Adakah anda bersetuju berikut adalah fungsi-fungsi yang meliputi tiga (3) skop utama yang perlu dilaksanakan oleh Pusat Operasi Keselamatan Siber:<br><br>(a) Pengurusan Pemantauan dan Pengumpulan Log<br>(b) Pengurusan Analisis<br>(c) Pengurusan dan Tindak Balas Insiden<br>(d) Pengurusan Forensik<br>(e) Pengurusan Operasi Asas Keselamatan Siber	✓		4
	D3-MPT20	Adakah anda bersetuju fungsi Pengurusan Pemantauan dan Pengumpulan Log perlu dilaksanakan oleh Pusat Operasi Keselamatan Siber?	✓		4
	D3-MPT21	Adakah anda bersetuju fungsi Pengurusan Analisis perlu dilaksanakan oleh Pusat Operasi Keselamatan Siber?	✓		4
	D3-MPT22	Adakah anda bersetuju fungsi Pengurusan dan Tindak Balas Insiden perlu dilaksanakan oleh Pusat Operasi Keselamatan Siber?	✓		4
	D3-MPT23	Adakah anda bersetuju fungsi Pengurusan Forensik perlu dilaksanakan oleh Pusat Operasi Keselamatan Siber?	✓		4
	D3-MPT24	Adakah anda bersetuju fungsi *Pengurusan Operasi Asas Keselamatan Siber perlu dilaksanakan oleh Pusat Operasi Keselamatan Siber?<br>**Melibatkan aktiviti seperti Imbasan Kelemah an, Ujian Penembusan dll.*	✓		4
	D3-MPT25	Adakah anda bersetuju kesemua fungsi berikut telah dilaksanakan oleh Pusat Operasi Keselamatan Siber?	✓		4
	D3-MPT26	Adakah anda bersetuju Pusat Operasi Keselamatan Siber yang melaksanakan ketiga-tiga skop Pemantauan, Analisis dan Tindak balas yang meliputi fungsi-fungsi di atas berupaya untuk melindungi organisasi daripada ancaman dan serangan siber?	✓		4

Bil.	Kod Soalan	Soalan	ULASAN PAKAR: Tandakan √ di ruang berkaitan — Relevan	ULASAN PAKAR: Tandakan √ di ruang berkaitan — Tidak Relevan	ULASAN PAKAR: Kepetingan Soalan — Pemberat (4/3/2/1)
**Sumber**		(Jacobs et al. 2013; Onwubiko 2015; Schinagl et al. 2015)			
	D3-MPT27	Dengan mengambilkira evolusi serangan siber masa kini, adakah anda bersetuju bahawa fungsi Pengurusan Kepintaran Ancaman juga perlu dilaksana kan oleh Pusat Operasi Keselamatan Siber?	✓		4
**Sumber**		(Al-Kaff et al. 2017; MDEC 2017; Miloslavskaya 2017; Rochford & MacDonald 2015; Schinagl et al. 2015)			
	D3-MPT28	Adakah anda bersetuju fungsi yang dijalankan di Pusat Operasi Keselamatan Siber adalah bergan tung kepada kemampuan kewangan organisasi?	✓		4
**Sumber**		(Ernst & Young 2014; Torress 2015)			

ULASAN PAKAR: BAHAGIAN D

Sila tandakan X bagi ruang yang di kehendaki.

- [X] Bersetuju
- [ ] Tidak bersetuju

Sekiranya tidak bersetuju, sila nyatakan kod soalan dan kenapa tidak bersetuju:

Soalan D1-MPT1 & MPT2 adalah soalan yang sama.
D3-MPT19-(C) Perkataan 'dan'

Catatan dan Cadangan Penambahbaikan (sekiranya ada):

D1-MPT1 boleh diolah utk fokus pada kemahiran teknikal spt protokol rangkaian komputer dan pemahaman kod komputer.

BAHAGIAN E : PENILAIAN TERHADAP PEMANTAUAN KESELAMATAN SIBER ORGANISASI

Soalan bahagian ini bertujuan untuk mendapakan pandangan responden mengenai strategi dan langkah keselamatan siber yang telah dilaksanakan di organisasi. Item-item yang terkandung dalam bahagian ini terdiri daripada **sepuluh (10) soalan** yang diukur menggunakan skala likert dengan pemilihan "Sangat Setuju", "Setuju", "Sederhana Setuju", "Tidak Setuju" dan "Sangat Tidak Setuju" sebagai pilihan jawapan.

**Bah.**	**Kod Soalan**	**Soalan**	**ULASAN PAKAR**		
			**Tandakan √ di ruang berkaitan**		**Kepetingan Soalan**
			**Relevan**	**Tidak Relevan**	**Pemberat (4/3/2/1)**
E	E-PEN1	Adakah anda bersetuju bahawa organisasi perlu menetapkan strategi keselamatan siber bagi melindungi infrastruktu r keselamatan maklumat?	√		4
	E-PEN2	Adakah anda bersetuju strategi keselamatan siber boleh dilaksanakan melalui kaedah pendekatan teknologi dan *bukan teknologi?<br><br>**Melibatkan aktiviti seperti Penilaian Risiko, NDA, ISMS dll.*	√		4
	E-PEN3	Adakah anda bersetuju kaedah pendekatan teknologi dan bukan teknologi bagi melindungi infrastruktur keselamatan maklumat telah dilaksanakan di organisasi anda?	√		4
**Sumber**		(Atif Ahmad et al. 2012)			
	E-PEN4	Adakah anda bersetuju bahawa kelemahan organisasi adalah berpunca daripada aspek manusia, proses dan teknologi?	√		4
	E-PEN5	Adakah anda bersetuju bahawa kelemahan organisasi daripada aspek manusia, proses dan teknologi boleh dieksploitasi o leh penggodam?	√		4
	E-PEN6	Adakah anda bersetuju bahawa kelemahan organisasi khususnya daripada aspek manusia adalah antara faktor yang menjadi punca sesuatu serangan siber?	√		4
**Sumber**		(Askarifar et al. 2018; Bock et al. 2017; Ey 2017; FireEye 2016; Kaspersky Lab 2017; Lee et al. 2016; Sharma & Verma 2017; Yeoman & Findlay 2017)			
	E-PEN7	Adakah anda bersetuju bahawa organisasi perlu bersiap sedia dalam menangani ancaman dan serangan siber dengan m elaksanakan langkah balas yang bersesuaian?	√		4
**Sumber**		(Bendovschi 2015; Gandhi et al. 2011; Uma & Padmavathi 2013)			

Bah.	Kod Soalan	Soalan	ULASAN PAKAR		
			Tandakan √ di ruang berkaitan		Kepetingan Soalan
			Relevan	Tidak Relevan	Pemberat (4/3/2/1)
	E-PEN8	Adakah anda bersetuju bahawa terdapat tiga (3) peringkat keselamatan iaitu Kawalan Pencegahan, Kawalan Pengesanan dan Kawalan Pembetulan yang perlu dilaksanaka n di organisasi?	√		4
	E-PEN9	Adakah anda bersetuju bahawa tiga (3) peringkat keselamatan iaitu Kawalan Pencegahan, Kawalan Pengesanan dan Kawalan Pembetulan telah dilaksanakan di organisasi anda?	√		4
	E-PEN10	Adakah anda bersetuju bahawa tiga (3) peringkat keselamatan iaitu Kawalan Pencegahan, Kawalan Pengesanan dan Kawalan Pembetulan ini boleh dilaksanakan oleh Pusat Operasi Keselamatan Siber?	√		4
**Sumber**		(Atif Ahmad et al. 2012; Bendovschi 2015)			

ULASAN PAKAR: BAHAGIAN E

Sila tandakan X bagi ruang yang di kehendaki.

[X] Bersetuju

[ ] Tidak bersetuju

Sekiranya tidak bersetuju, sila nyatakan kod soalan dan kenapa tidak bersetuju:

Perkataan 'pembetulan' dan perkataan 'langkah balas'

Catatan dan Cadangan Penambahbaikan (sekiranya ada):

- Ganti perkataan 'pembetulan' kpd 'pemulihan.'
- Ganti perkataan 'langkah balas' kepada 'tindak balas'.

3.0 PENGESAHAN ULASAN PAKAR

Setelah meneliti Kaji Selidik Pengenalpastian Faktor Kejayaan Pelaksanaan Pusat Operasi Keselamatan Siber di Persekitaran Sektor Awam Malaysia, dengan ini saya mengesahkan kandungan kaji selidik yang disediakan oleh:

Nama Pelajar	:	Maziana Abd Majid
No Matrik	:	GP04714
Kursus	:	Sarjana Keselamatan Siber, UKM
Tajuk Kajian	:	Pengenalpastian Faktor Kejayaan Pelaksanaan Pusat Operasi Keselamatan Siber di Persekitaran Sektor Awam Malaysia
Penyelia	:	Dr. Khairul Akram bin Zainol Ariffin

dengan ulasan seperti berikut:

Penyelidikan yang baik dan merangkumi semua aspek penting utk sesebuah SOC.

Sekian, harap maklum.

Tanda Tangan	:	[illegible]
Nama	:	[illegible]
Cop Jawatan	:	
Fakulti / Jabatan	:	Forensik Digital
Tarikh	:	25-4-2019

Example of Experts Evaluation on the Model.

Note: it is in malay language

LAMPIRAN A BORANG PENGESAHAN DAN ULASAN MODEL KAJIAN OLEH PAKAR I

Model Keperluan Asas Pembangunan dan Pelaksanaan Pusat Operasi Keselamatan Siber – *Security Operation Centre* (SOC) Bagi Persekitaran Sektor Awam Malaysia Yang Meliputi Faktor Manusia, Proses Dan Teknologi

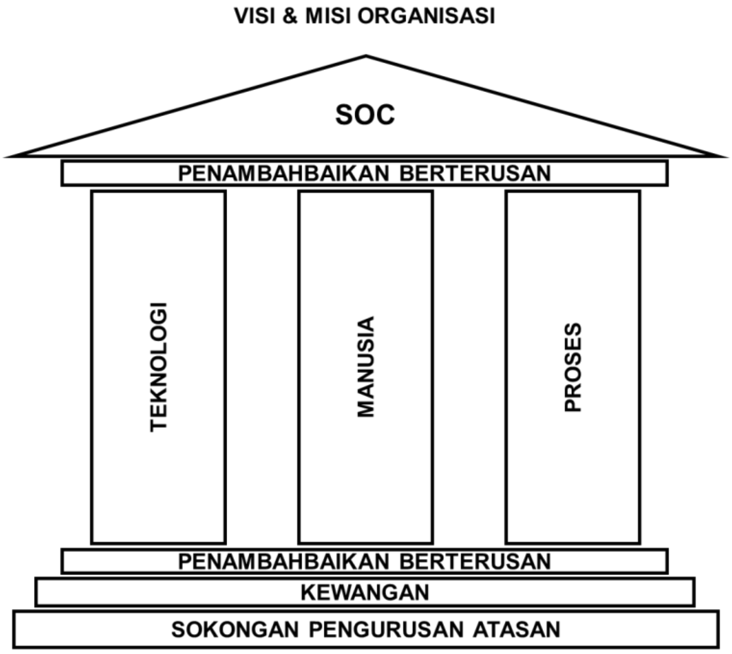

Sila berikan idea dan cadangan penambahbaikan bagi faktor yang disenaraikan di bawah:

Bil.	Perkara / Faktor	Ulasan / Cadangan Penambahbaikan
1	Manusia	Setuju
2	Proses	Setuju
3	Teknologi	Setuju
4	Lain - lain	• Bersetuju dengan model secara keseluruhannya<br>• Model perlu mengenal pasti tadbir urus yang jelas

Perakuan:

Dengan ini, saya mengesahkan penilaian terhadap model yang telah diberikan:

Nama : ______________________________

Agensi : ______________________________

Jawatan : ______________________________

Cop Jabatan : Infrastruktur & Keselamatan ICT
MAMPU, Jabatan Perdana Menteri

10 Februari 2020

BORANG PENGESAHAN DAN ULASAN MODEL KAJIAN OLEH PAKAR II

Model Keperluan Asas Pembangunan dan Pelaksanaan Pusat Operasi Keselamatan Siber – *Security Operation Centre* (SOC) Bagi Persekitaran Sektor Awam Malaysia Yang Meliputi Faktor Manusia, Proses Dan Teknologi

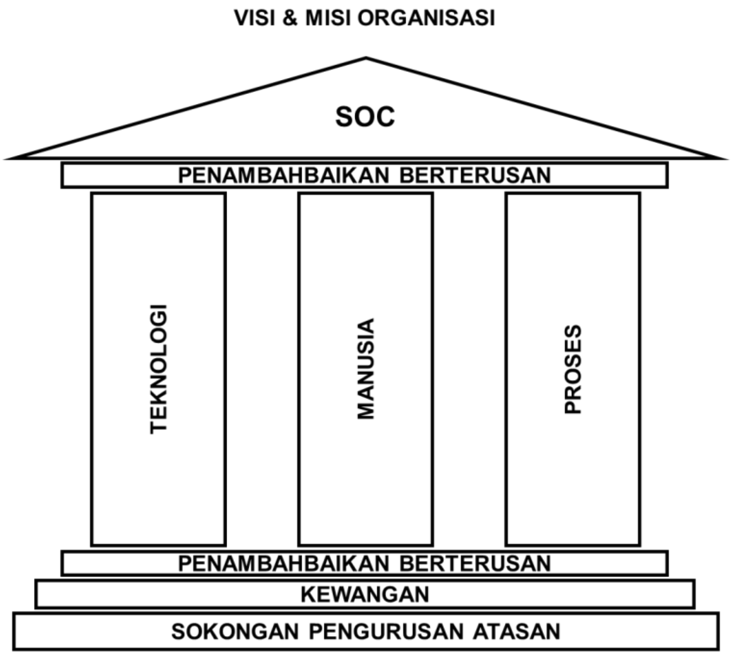

Sila berikan idea dan cadangan penambahbaikan bagi faktor yang disenaraikan di bawah:

Bil.	Perkara / Faktor	Ulasan / Cadangan Penambahbaikan
1	Manusia	Setuju
2	Proses	Setuju
3	Teknologi	Setuju
4	Lain - lain	▪ Bersetuju dengan model yang dibangunkan<br>▪ Model yang efektif<br>▪ Model perlu mengenal pasti saluran *Intelligance Exchange* di antara SOC

Perakuan:

Dengan ini, saya mengesahkan penilaian terhadap model yang telah diberikan:

Nama :

Agensi : [illegible]

Bahagian Perundingan ICT
MAMPU, Jabatan Perdana Menteri

Jawatan :

10/2/2020

Cop Jabatan :
